# The mRNA export factor UAP56 is required for dendrite and synapse pruning via actin regulation in *Drosophila*

**DOI:** 10.1242/jcs.264770

**Published:** 2026-05-11

**Authors:** Samuel Matthew Frommeyer, Ulrike Gigengack, Sandra Rode, Matthew Davies, Neele Wolterhoff, Sebastian Rumpf

**Affiliations:** Multiscale Imaging Center and Institute for Neurobiology, University of Münster, Röntgenstrasse 16, 48149 Münster, Germany

**Keywords:** Dendrite, Synapse, Pruning, UAP56, Actin, Cofilin

## Abstract

Neurite and synapse pruning are conserved mechanisms that adapt neuronal circuitry to different developmental stages. *Drosophila* sensory c4da neurons prune their larval dendrites and their presynaptic terminals during metamorphosis using a gene expression program that is induced by the steroid hormone ecdysone and involves post-transcriptional regulation pathways. Here, we show that loss of the helicase UAP56, an important mediator of nuclear mRNA export, causes strong dendrite and presynapse pruning defects. Loss of UAP56 is linked to actin regulation, as it causes defects in the ecdysone-induced expression of the actin-severing enzyme Mical during metamorphosis and actin accumulation at pruning presynapses. In support of an important role of actin regulation during presynaptic pruning, we find that cofilin is required for this process. Our findings highlight the role of post-transcriptional regulation in neuronal remodeling and identify an actin disassembly factor required for presynapse pruning.

## Teaser

**Summary:** Loss of the mRNA export factor UAP56 leads to dendrite and presynapse pruning defects via defects in actin disassembly pathways in *Drosophila*.

## INTRODUCTION

Neurite and synapse pruning, the regulated degeneration of neuronal connections without loss of the parent neuron, serve to specify neuronal circuits and to remove developmental intermediates during neuronal development (Riccomagno and Kolodkin, 2015; Rumpf et al., 2017). Dysregulation of pruning pathways has been linked to developmentally caused neurological syndromes such as autism spectrum disorders and schizophrenia (Tang et al., 2014; Howes and Onwordi, 2023). *Drosophila* is an ideal model in which to study the cell biological mechanisms underlying pruning. For example, the peripheral sensory class IV dendritic arborization (c4da) neurons prune both their larval sensory dendrites and their central presynapses in the ventral nerve cord (VNC) during metamorphosis (Kuo et al., 2005; Williams and Truman, 2005; Furusawa et al., 2023). Dendrite pruning is induced by a pulse of the steroid hormone ecdysone at the onset of metamorphosis, which induces the expression of pruning factors such as the transcription factor Sox14 and the actin-severing enzyme Mical (Kirilly et al., 2009). To facilitate pruning, ecdysone signaling also leads to upregulation of the ubiquitin-proteasome system (Chew et al., 2021) and changes in neuronal metabolism (Marzano et al., 2021). A key target during large-scale pruning is the microtubule cytoskeleton, which is locally disassembled in dendrites (Herzmann et al., 2017, 2018; Rumpf et al., 2019; Davies et al., 2025), eventually leading to mechanical tearing of the whole dendrite close to the cell body (Krämer et al., 2023). While c4da neuron axons stay intact during metamorphosis (Kuo et al., 2005), their presynaptic terminals are pruned in an ecdysone- and ubiquitin-dependent manner (Furusawa et al., 2023). However, to date little is known about cytoskeletal regulation during presynapse pruning.

As c4da neuron pruning is induced by changes in gene expression, several post-transcriptional control mechanisms have been implicated in pruning, including splicing (Rumpf et al., 2014; Rode et al., 2017) and translation control (Rode et al., 2018). Here, specific requirements in the translation machinery during neuronal remodeling could be linked to differential activity of the Target of Rapamycin (TOR) pathway during development (Wong et al., 2013; Sanal et al., 2023).

Nuclear mRNA export occurs after, and depends on, mRNA splicing. Here, a set of dedicated export factors, including the DEXD box ATPase UAP56 (also known as HEL25E in *Drosophila*), the THO complex and the protein Alyref (Ref1 in *Drosophila*) are co-transcriptionally loaded onto nascent messenger ribonucleoprotein (mRNP) complexes. En route to the nuclear pore, this mRNA–UAP56–THO–Aly complex (also called TREX) is disassembled in a step-wise manner. The UAP56 ATPase is activated by the TREX-2 complex and UAP56 is released from the mRNP in the last step at the nuclear pore, where the export factor NXF1 (Sbr in *Drosophila*) is loaded and the mRNA is exported (Xie and Ren, 2019; Hohmann et al., 2025). While this pathway is thought to be required for the export of most or all mRNAs, loss of single mRNA export factors can lead to surprisingly specific phenotypes. For example, in *Caenorhabditis elegans* and mouse dopaminergic neurons, the THO complex is particularly important for export of mRNAs encoding synaptic proteins (Maeder et al., 2018). Furthermore, congenital mutations in the human UAP56 homolog DDX39B were recently shown to cause neurodevelopmental defects (Booth et al., 2025).

Here, we show that UAP56 is required for c4da neuron dendrite and presynapse pruning in *Drosophila*. While important pruning pathways such as the ubiquitin-proteasome system and microtubule organization appear unaffected by loss of UAP56, cytoplasmic levels of *Mical* mRNA are reduced, and Mical protein expression is delayed, indicating that *Mical* mRNA export is strongly dependent on UAP56. At the presynapse, loss of UAP56 causes pruning defects concomitant with actin accumulation. In contrast to dendrite pruning, where Mical is important for actin disassembly, presynapse pruning depends on the actin disassembly factor cofilin. Thus, our results describe a neurodevelopmental function for an mRNA export factor involved in human neurological disease and new factors involved in presynapse pruning.

## RESULTS

### The mRNA export factor UAP56 is required for c4da neuron dendrite pruning

**Fig. 1.**
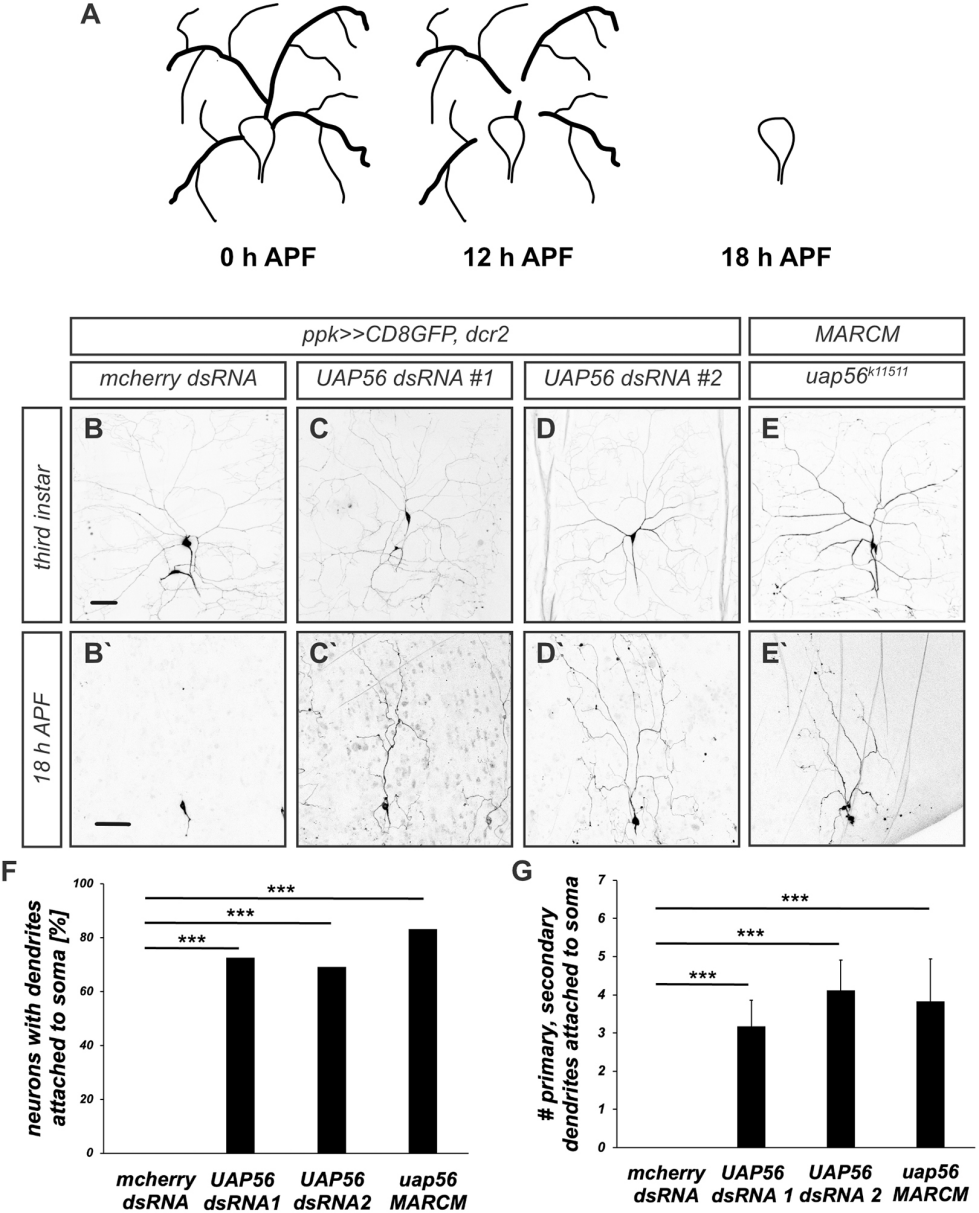
**UAP56 is required for c4da neuron dendrite pruning.** (A) Schematic timecourse of c4da neuron dendrite pruning. (B-E′) C4da neurons were labeled by CD8::GFP expression under *ppk-GAL4* or tdtomato expression in MARCM clones and visualized at the third instar larval stage (B-E) or at 18 h APF (B′-E′). (B,B′) Control c4da neurons expressing mCherry dsRNA. (C,C′) C4da neurons expressing UAP56 dsRNA#1. (D,D′) C4da neurons expressing UAP56 dsRNA#2. (E,E′) Homozygous *uap56^k11511^* c4da neuron MARCM clones. (F) Penetrance of the pruning defects shown in B′-E′. *n*=24-25 neurons, ***P<0.0005 (two-tailed Fisher's Exact test). (G) Severity of pruning defects as assessed by the number of attached primary and secondary dendrites, as shown in B′-E′. *n*=24-25 neurons. ***P<0.0005 (Wilcoxon's test). Error bars represent s.e.m. Scale bars: 50 µm (in B, for B-E; in B′ for B′-E′).

C4da neurons prune their larval dendrites within the first 18 h after puparium formation (h APF) (Fig. 1A-B′). To gain further insight into post-transcriptional regulation of dendrite pruning, we conducted an RNAi screen for mRNA-related factors. We used the c4da-specific driver *ppk-GAL4* to express 103 available dsRNA lines against 64 genes that had previously been linked to splicing in S2 cells (Park et al., 2004) (Table S1). This approach identified three lines each directed against eIF4A (Rode et al., 2018) and UAP56. C4da neurons expressing UAP56 dsRNA exhibited relatively normal, if slightly reduced, dendritic arbors at the third instar stage (Fig. 1C), even though they displayed lower levels of the CD8::GFP reporter used to visualize c4da neurons (see higher background in Fig. 1C,C′). At 18 h APF, neurons expressing UAP56 dsRNA showed strong dendrite pruning defects (Fig. 1C′,F,G). We confirmed these results using a second dsRNA line directed against UAP56 (Fig. 1D,D′,F,G). To verify the UAP56 knockdown result by an independent method, we performed mosaic analysis with a repressible marker (MARCM) with *uap56^k11511^*, a lethal P element insertion in the 5′ untranslated region (UTR) of the *uap56* locus, and found that homozygous *uap56* mutant c4da neurons also displayed strong dendrite pruning defects at 18 h APF (Fig. 1E-G). As UAP56 is a regulator of mRNA export from the nucleus, we tested whether downregulation of other mRNA export factors also caused dendrite pruning defects. However, knockdown of the THO complex components THO2 and THOC5 as well as of NXF1 did not affect dendrite pruning (Fig. S1). We conclude that c4da neuron dendrite pruning is particularly sensitive to loss of UAP56.

### Role of UAP56 ATPase activity

**Fig. 2.**
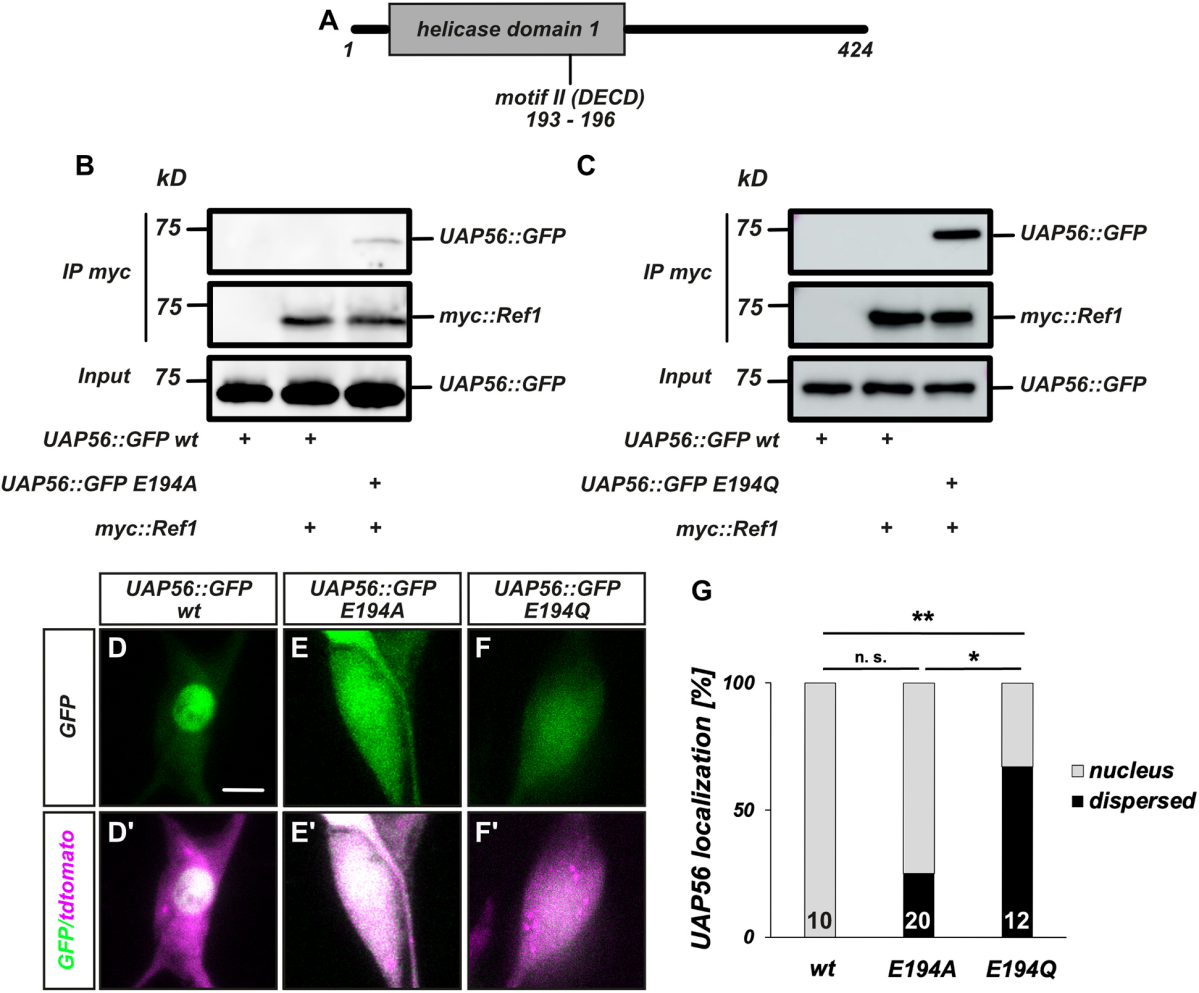
**A UAP56 ATPase mutation affects its protein interactions and localization.** (A) Schematic indicating the position of the UAP56 ATPase domain and the DECD motif. (B,C) Co-immunoprecipitation between UAP56 variants and Ref1. GFP-tagged UAP56 constructs [B: wild type (wt) and E194A; C: wt and E194Q] were co-expressed with myc::Ref1 in S2 cells, and precipitated using anti-Myc antibodies. Shown are bound fractions (top) and input fractions for UAP56::GFP (bottom). The position of a molecular weight marker is indicated to the left. (D-G) Localization of GFP-tagged UAP56 variants in c4da neurons. The indicated UAS transgenes were expressed in *uap56* MARCM clones and visualized by confocal microscopy. Shown are the GFP channel (top) or GFP merged with the cell marker tdtomato (bottom). (D,D′) UAP56 wt. (E,E′) UAP56 E194A. (F,F′) UAP56 E194Q. (G) Penetrance of nuclear versus dispersed cellular distribution patterns as shown in D-F′. *n*=10-20 neurons. *P<0.05, **P<0.005 (Fisher's Exact test). n. s., not significant. Scale bar: 10 µm (D).

UAP56 is thought to coordinate the transition of mRNP particles from transcription sites to the nuclear pore, and its ATPase activity is involved in the disassembly of a late nuclear export intermediate hnRNP complex that includes Ref1 (Xie and Ren, 2019; Hohmann et al., 2025). UAP56 possesses a Walker A/B type helicase ATPase domain, where a DECD sequence (called motif II, amino acids 193-196, corresponding to 215-218 in yeast Sub2 and 195-198 in human UAP56) in the Walker B domain is important for ATP hydrolysis (Shen et al., 2007) (Fig. 2A). ATPase activity in the yeast homolog Sub2 is required for viability (Saguez et al., 2013), but functional data from cell types of multicellular organisms, such as postmitotic neurons, are missing. To specifically inhibit the UAP56 ATPase, we disrupted the DECD sequence by mutating the glutamate in the motif to either alanine or glutamine, thus generating the variants UAP56^E194A^ (DECD to DACD) and UAP56^E194Q^ (DECD to DQCD). Mutation of this residue in DEAD box proteins such as eIF4A can generate strong dominant-negatives (Rode et al., 2018). To test whether the ATPase affects UAP56 protein interactions, we next performed co-immunoprecipitation experiments with the known UAP56 binding partner Ref1 in S2 cells. While wild-type UAP56::GFP could not be detected in precipitates of Myc-tagged Ref1 (likely due to fast disassembly in the S2 cell lysate), both UAP56::GFPE194A (Fig. 2B) and UAP56::GFPE194Q (Fig. 2C) could be co-precipitated. To test whether this result was a consequence of an inherent ‘stickyness’ of the UAP56 ATPase mutants, we tested whether we could co-immunoprecipitate THOC7, a subunit of the THO complex, with the UAP56 variants. In keeping with the notion that the THO complex is released from UAP56 independently of its ATPase (Hohmann et al., 2025), we could not detect tagged THOC7 in precipitates of wild-type UAP56 or in those with UAP56^E194Q^ (Fig. S2). This indicates that UAP56 E194 mutants are unlikely to undergo nonspecific interactions.

As little is known about the potential effects of the ATPase on UAP56 cellular localization, we next analyzed this by expressing the corresponding GFP-tagged variants in *uap56* c4da neuron MARCM clones. Wild-type UAP56::GFP localized exclusively to the nucleus (Fig. 2D,D′,G). The UAP56^E194A^ also localized to the nucleus, but its levels in the cytoplasm were increased, and it was evenly distributed between nucleus and cytoplasm in a subset of neurons (Fig. 2E,E′,G). This effect was even more pronounced with the UAP56^E194Q^ mutant, which was evenly distributed between nucleus and cytoplasm in most analyzed neurons (Fig. 2F,G), indicating that an active ATPase is required to maintain UAP56 localization in the nucleus.

**Fig. 3.**
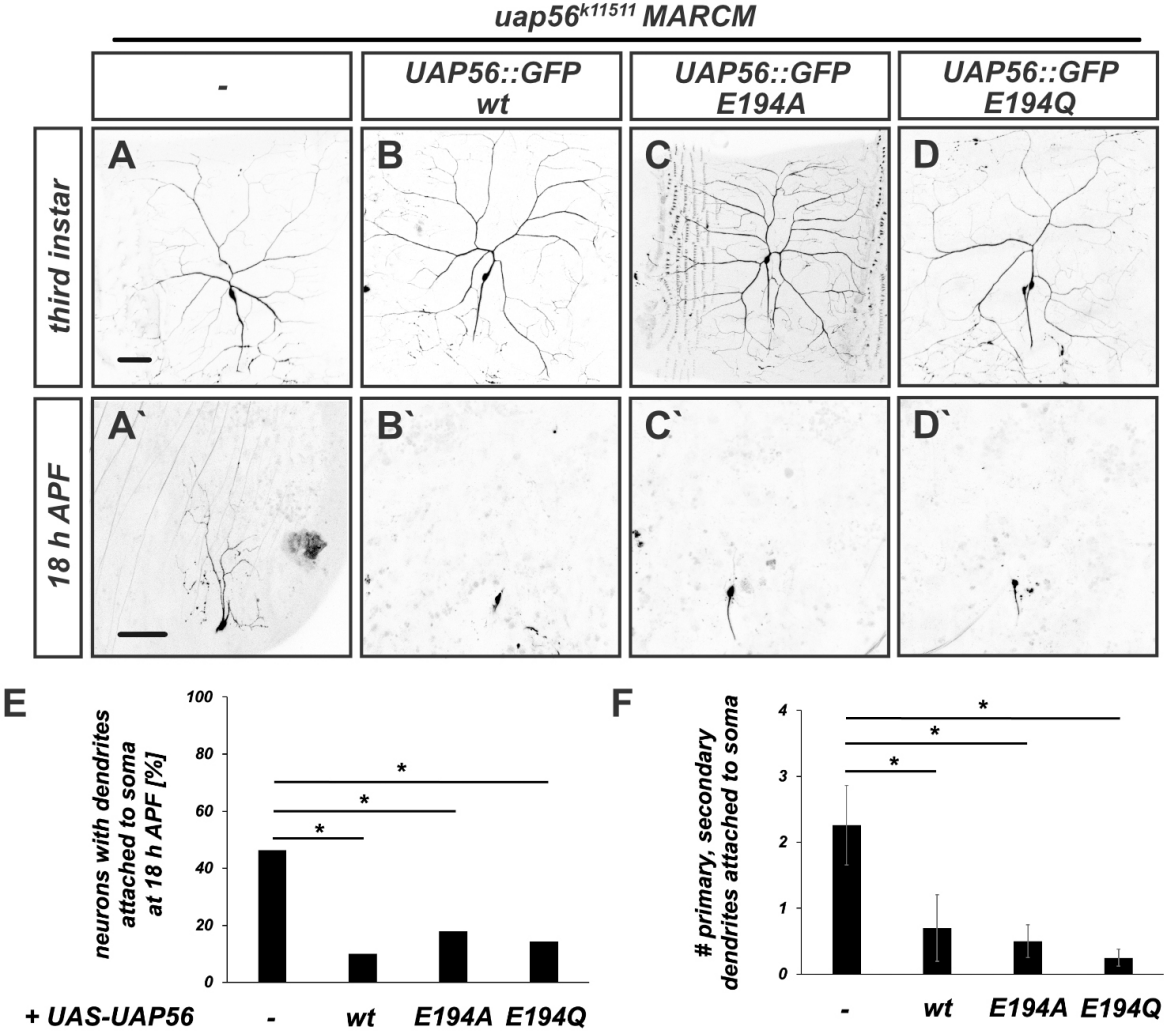
**Evidence that the UAP56 ATPase is not required for dendrite pruning.** (A-D′) The indicated UAP56::GFP constructs were expressed in *uap56^k11511^* c4da neuron MARCM clones and morphology was assessed at the third instar (A-D) or at 18 h APF (A′-D′). (A,A′) *uap56^k11511^* c4da neuron MARCM clones. (B,B′) *uap56^k11511^* c4da neuron MARCM clones expressing UAP56::GFP wt. (C,C′) *uap56^k11511^* c4da neuron MARCM clones expressing UAP56::GFP E194A. (D,D′) *uap56^k11511^* c4da neuron MARCM clones expressing UAP56::GFP E194Q. (E) Penetrance of c4da neuron dendrite pruning defects, as shown in A′-D′. *n*= 25, 20, 28, 28 neurons. *P<0.05 (Fisher's Exact test). (F) Severity of c4da neuron dendrite pruning defects as assessed by the number of attached primary and secondary dendrites, as shown in A′-D′. *P<0.05 (Wilcoxon's test). Error bars represent s.e.m. Scale bars: 50 µm (in A, for A-D; in A′, for A′-D′).

We next investigated whether UAP56 ATPase is necessary for dendrite pruning. To this end, we expressed UAP56::GFP wt, UAP56::GFP^E194A^ or UAP56::GFP^E194Q^ in *uap56^k11511^* MARCM clones and tested whether they can rescue the pruning defects at 18 h APF. Interestingly, both wild-type UAP56 (Fig. 3A-B′,E,F) and the two UAP56 E194 mutants (Fig. 3C-F) rescued the pruning defects to similar degrees. Thus, our data suggest that UAP56 ATPase activity is required for disassembly of a UAP56–Ref1 complex and for UAP56 nuclear localization in c4da neurons, but not for its function during c4da neuron dendrite pruning.

### Loss of UAP56 does not affect microtubule regulation or the ubiquitin system

**Fig. 4.**
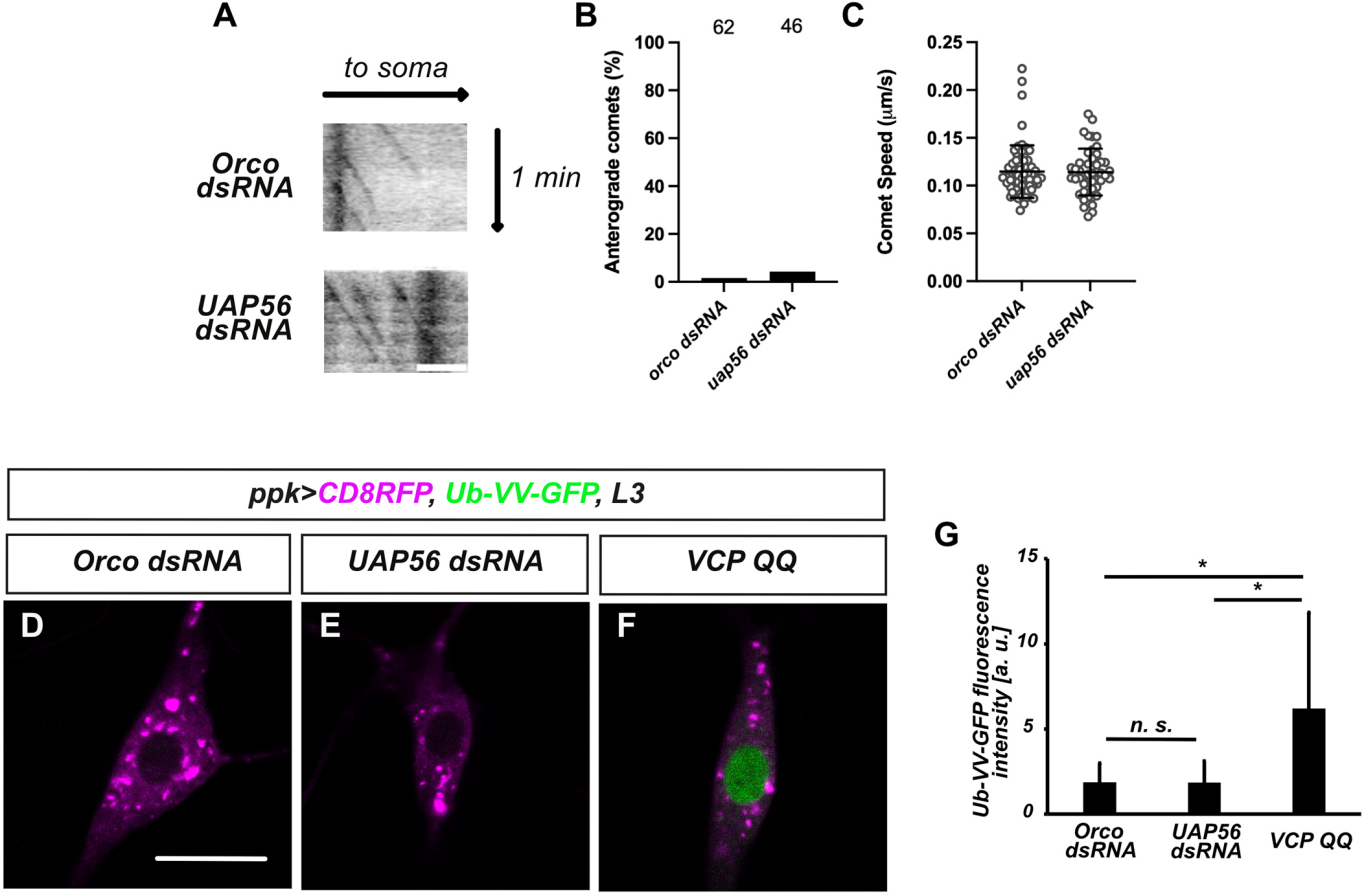
**UAP56 loss does not affect microtubule organization or ubiquitin-mediated protein degradation.** (A-C) Microtubule orientation and growth speed were determined using EB1::GFP. (A) Dendritic EB1::GFP kymographs from control c4da neurons expressing Orco dsRNA (top) or UAP56 dsRNA (bottom). Upper arrow indicates direction of soma (retrograde). (B) Percentage of anterograde comets for the experiment shown in A. Numbers of comets are indicated in the graph. Fisher's Exact test, *P*-value not significant. (C) Comet speed for the experiment shown in A. *n*=62 and 46 (Orco dsRNA and UAP56 dsRNA, respectively). Data are mean±s.d. Wilcoxon's test, *P*-value not significant. (D-G) Use of a Ub-VV-GFP reporter to assess potential effects on ubiquitin-proteasome system function in c4da neurons. (D-F) Reporter GFP fluorescence together with CD8::RFP to label the neuron. (D) Control c4da neuron expressing Orco dsRNA. (E) C4da neuron expressing UAP56 dsRNA. (F) C4da neuron expressing dominant-negative VCP QQ. (G) Quantification of Ub-VV-GFP fluorescence intensity for the experiment shown in D-F. *n*=9-11 neurons. Data are mean±s.d., *P<0.05 (two-tailed unpaired Student's *t*-test). n.s., not significant. Scale bar: 5 µm (A); 10 µm (D).

To begin to address how UAP56 deficiency affects c4da neuron dendrite pruning, we next assessed the effects of UAP56 knockdown on known important pruning pathways. C4da neuron dendrite pruning requires a coordinated disassembly of dendritic microtubules that depends on their uniform orientation with their plus ends towards the soma (‘plus end-in’) (Rumpf et al., 2019). To test for the organization of the dendritic microtubule cytoskeleton, we live-imaged GFP-tagged EB1, a plus end-binding protein that can be used to track the growth direction of microtubules. EB1-bound plus ends of growing microtubules can be detected in dendrites as moving puncta (also called ‘EB1 comets’). EB1 comet movement can then be analyzed in kymograph analyses, which show moving EB1 puncta as diagonal lines (Fig. 4A). This showed that neither the directionality (Fig. 4B) nor the speed (Fig. 4C) of dendritic EB1 comets were altered in neurons expressing UAP56 dsRNA compared to neurons expressing a control dsRNA, indicating normal dendritic microtubule organization.

C4da neuron dendrite pruning also requires the ubiquitin-proteasome system (Kuo et al., 2005; Rumpf et al., 2011; Wong et al., 2013). To assess global ubiquitin-proteasome system function, we used a ubiquitin fusion degradation (UFD) reporter. Here, ubiquitin is N-terminally fused to GFP, and the C-terminal glycine residues of ubiquitin are changed to valine, which renders the fusion noncleavable by deubiquitylating enzymes and thus targets the reporter for rapid proteasomal degradation. Ub-VV-GFP was undetectable in c4da neurons expressing both control and UAP56 dsRNAs (Fig. 4D,E,G). As a proof of reporter functionality, we expressed VCP QQ, a dominant-negative version of the UPS chaperone VCP (Rumpf et al., 2011), which caused reporter stabilization (Fig. 4F,G). To independently validate the UAP56 knockdown result, we also stained c4da neurons expressing control or UAP56 dsRNAs with antibodies against ubiquitin. This also showed no obvious difference between control and UAP56 knockdown neurons (Fig. S3). Thus, two important cellular pruning pathways are not affected by loss of UAP56.

### Loss of UAP56 affects Mical expression

**Fig. 5.**
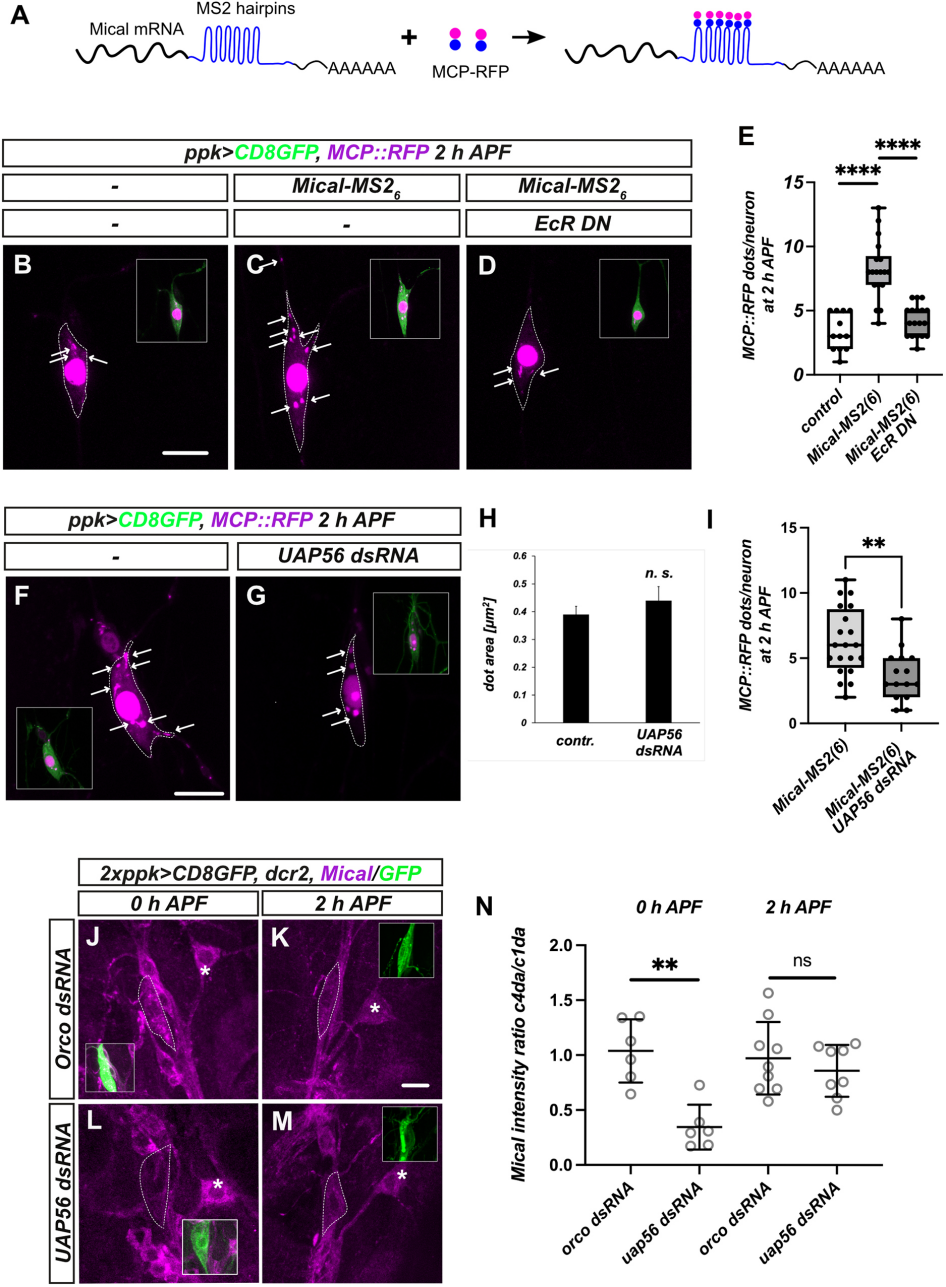
**Effect of UAP56 on Mical mRNA and protein expression.** (A) Schematic of the MS2-MCP system. *Mical* mRNA was labeled by knock-in of the MS2 sequence in the 3′UTR, enabling visualization by expression of MCP::RFP. (B-E) Characterization of *Mical-MS2_6_*. MCP::RFP was expressed in c4da neurons of the indicated genotypes and visualized at 2 h APF. Cytoplasmic MCP::RFP puncta are labeled by arrows. Insets show CD8::GFP expression in c4da neurons. (B) Control c4da neuron in a wild-type background. (C) Control c4da neuron in the *Mical-MS2_6_* background. (D) C4da neuron expressing dominant-negative EcR-DN in the *Mical-MS2_6_* background. (E) Number of cytoplasmic MCP::RFP puncta for the experiment shown in B-D. Boxes represent first quartiles and the median is indicated. Whiskers show s.d. *n*=12, 16, 18 neurons (left to right). ***P<0.0005 (Wilcoxon's test). (F-I) Effect of UAP56 knockdown on *Mical-MS2_6_* particles at 2 h APF. (F) Control c4da neuron in the *Mical-MS2_6_* background. (G) C4da neuron expressing UAP56 dsRNA in the *Mical-MS2_6_* background. (H) Quantification of MCP::RFP dot area for the experiment shown in F,G. *n*=12 neurons each, 58 and 43 dots, respectively. n. s., not significant (*P*>0.05, two-tailed unpaired Student's *t*-test). Values represent mean±s.e.m. (I) Quantification of cytoplasmic MCP::RFP puncta for the experiment shown in F,G. *n*=20 and 15 neurons (left to right). **P<0.005 (Wilcoxon's test). (J-N) Effect of UAP56 knockdown on Mical protein expression. Mical was visualized by immunofluorescence at 0 or 2 h APF. The indicated dsRNAs were driven by *ppk-GAL4*. c4da neurons are indicated by dashed lines (insets show GFP label). (J) Control c4da neuron expressing Orco dsRNA at 0 h APF. (K) Control c4da neuron at 2 h APF. (L) C4da neuron expressing UAP56 dsRNA at 0 h APF. (M) C4da neuron expressing UAP56 dsRNA at 2 h APF. (N) Quantification of Mical expression levels for the experiment shown in J-M. Mical expression in c4da neurons was normalized to expression levels in neighboring c1da neurons not expressing dsRNA (asterisks). *n*=6 neurons (0 h APF) or *n*=8-9 neurons (2 h APF). Data are mean±s.d. ns, not significant, **P<0.005 (two-tailed unpaired Student's *t*-test). ns, not significant. Scale bars: 10 µm (B,F,K).

The ecdysone-Sox14-MICAL pathway is an important transcriptional pathway during dendrite pruning (Kirilly et al., 2009). Sox14 could be detected in c4da neurons expressing UAP56 dsRNA at both 0 and 2 h APF (Fig. S4). The Sox14 target gene *Mical* encodes a large actin-severing protein of 4723 amino acids (from a 15 kb mRNA) (Terman et al., 2002). Because of the large size of its mRNA, we reasoned that *Mical* gene expression could be particularly sensitive to loss of export factors such as UAP56. To test whether loss of UAP56 affects *Mical*, we sought to visualize *Mical* mRNA in c4da neurons. The MS2-MCP system (Bertrand et al., 1998) and similar systems have previously been used to semi-quantitatively assess nuclear mRNA export in yeast (Smith et al., 2015) and dendritic mRNA transport in c4da neurons (Brechbiel and Gavis, 2008). Here, several copies of the MS2 sequence are introduced into an mRNA of interest and a fluorescently tagged version of the MS2-binding protein MCP is co-expressed with the tagged mRNA. MCP contains a nuclear localization signal and localizes in the nucleus, except when it is exported as part of an MS2-tagged mRNA particle. We introduced six copies of the MS2 sequence into the Mical 3′UTR using a CRISPR-based strategy (Zhang et al., 2014) (Fig. 5A, Fig. S5). We then expressed an RFP-tagged version of the MS2-binding protein MCP in c4da neurons. Larval c4da neurons, which do not express Mical, contained approximately ten small cytoplasmic MCP::RFP dots in the cell body irrespective of background (control or *Mical*-MS2_6_), likely due to strong MCP::RFP expression under *ppk-GAL4* at this stage (Fig. S5). At 2 h APF, control c4da neurons without *Mical*-MS2_6_ displayed only three or four relatively dim puncta in the cytosol (Fig. 5B,E), but approximately eight bright MCP::RFP puncta could be seen in homozygous *Mical*-MS2_6_ animals at this stage (Fig. 5C,E). To ascertain that these dots represented tagged *Mical* mRNA, we expressed EcR DN, a dominant-negative version of the ecdysone receptor that prevents Mical expression. This reduced the number of MCP::RFP puncta to background levels (Fig. 5D,E).

Having thus validated our system, we next examined whether UAP56 loss has an effect on *Mical* mRNA. Compared to control neurons, c4da neurons expressing UAP56 dsRNA had a reduced number of cytoplasmic MCP::RFP puncta (Fig. 5F,G,I). MCP-labeled particles in this system might contain multiple mRNA molecules (Smith et al., 2015). To test whether the MCP-labeled particles themselves were altered in UAP56 knockdown samples, we also measured the dot area in control and UAP56 knockdown samples and found that it was not significantly changed (Fig. 5H). Thus, our data are consistent with the idea that *Mical* mRNA depends on UAP56 for its nuclear export.

To verify that loss of UAP56 affects Mical expression, we next assessed Mical protein levels in control and UAP56 knockdown c4da neurons by immunofluorescence. Mical expression in control neurons was detectable as early as the white pupal stage (0 h APF) and persisted beyond 2 h APF (Fig. 5J,K,N). In neurons expressing UAP56 dsRNA, Mical protein could not be detected at 0 h APF (Fig. 5L,N). However, we detected almost normal levels of Mical protein at 2 h APF (Fig. 5M,N), indicating that UAP56 knockdown leads to a delay in Mical protein accumulation. Unfortunately, the strength of UAP56 dsRNA constructs was sensitive to titration, so we could not reliably assess whether exogenous Mical expression can rescue the UAP56 loss-of-function phenotype. However, loss of Mical expression is causal to the pruning defects of a number of other mutants (Kirilly et al., 2009; Rode et al., 2018; Wolterhoff et al., 2020), making it likely that UAP56 deficiency affects c4da neuron dendrite pruning at least in part by delaying Mical expression.

### UAP56 is required for c4da neuron presynapse pruning

**Fig. 6.**
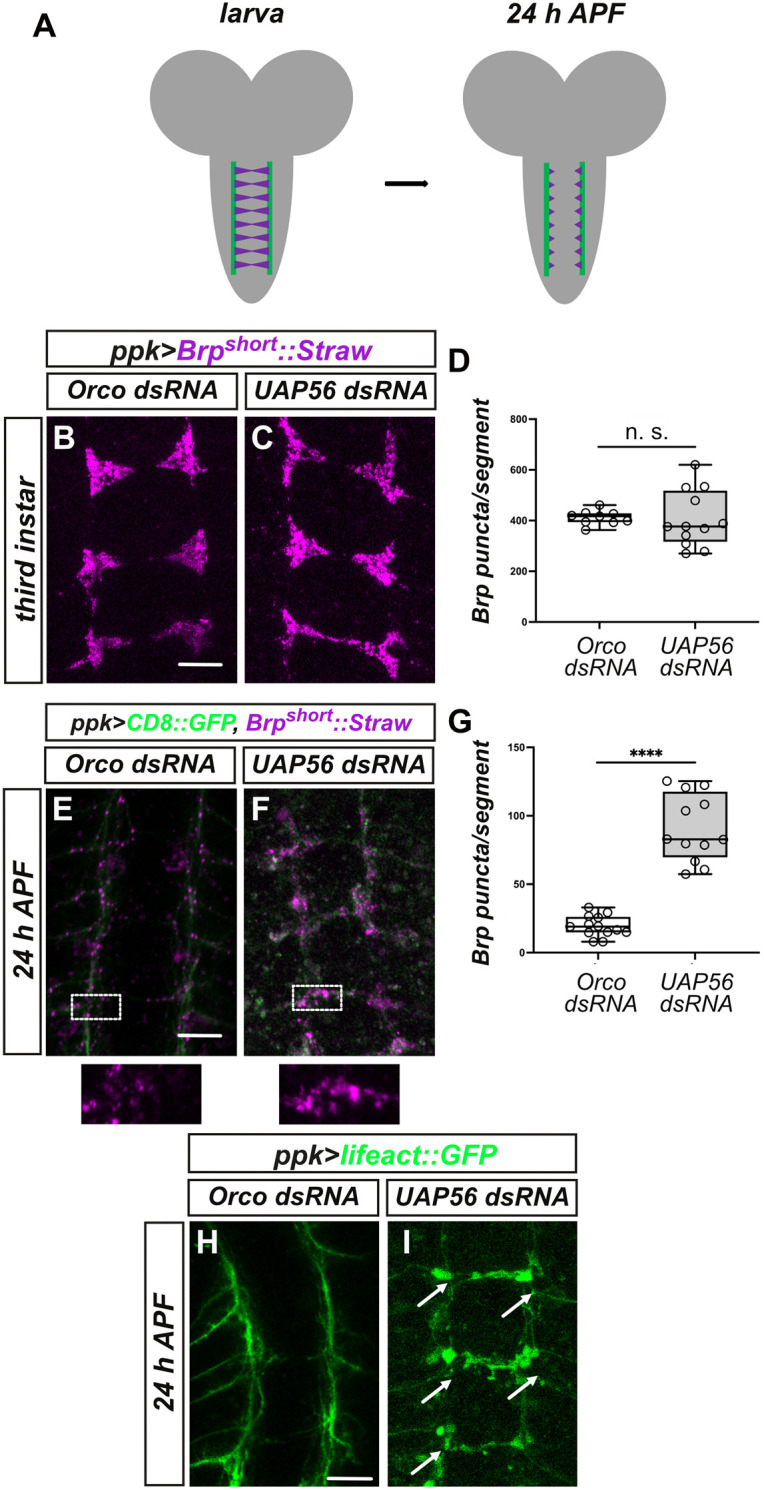
**Loss of UAP56 causes c4da neuron presynapse pruning defects.** (A) C4da neuron presynapse pruning during metamorphosis. Schematic shows the ladder-like segmental arrangements of c4da neuron axons (green) and presynaptic areas (magenta) in the VNC at the larval stage and at 24 h APF. (B-D) Effect of UAP56 loss on c4da neuron active zone number in third instar larvae (segments A3-A5). C4da neuron active zones in the VNC were labeled by expression of *Brp^short^::Strawberry* via *ppk-GAL4*. (B) Control c4da neurons expressing Orco dsRNA. (C) C4da neurons expressing UAP56 dsRNA. (D) Number of *Brp^short^::Strawberry* puncta in larvae. The average number of synaptic Brp puncta per segment (segments A3-A5) was determined for each animal using SynQuant. Box indicates inner quartiles, the median is indicated by a line. Whiskers represent s.d. *n*=9, 13 animals (Orco dsRNA and UAP56 dsRNA, respectively). n. s., not significant (unpaired *t*-test). (E,F) C4da neuron active zones at 24 h APF. Lower panels show magnified views of the *Brp^short^::Strawberry* signal in the boxed areas above. (E) Control animals expressing Orco dsRNA in c4da neurons. (F) Animals expressing UAP56 dsRNA. (G) Number of *Brp^short^::Strawberry* puncta at 24 h APF. *n*=14, 12 animals (Orco dsRNA and UAP56 dsRNA, respectively). ****P<0.0001 (unpaired *t*-test). (H,I) Loss of UAP56 causes actin accumulation at presynaptic sites during the pupal stage. F-actin was labeled using *UAS-lifeact::GFP* expressed under *ppk-GAL4*, and c4da neuron axonal endings in the VNC in segments A3-A5 were visualized at 24 h APF. (H) Control c4da neurons expressing Orco dsRNA. (I) C4da neurons expressing UAP56 dsRNA. Actin accumulations are indicated by arrows. Scale bars: 10 µm (B,F,H).

While c4da neuron axons persist during metamorphosis (Kuo et al., 2005), their central presynapses in the VNC are also pruned in an ecdysone-dependent manner with a similar timecourse as c4da neuron dendrites (Furusawa et al., 2023) (Fig. 6A). To fully explore the extent of the defects caused by loss of UAP56, we next assessed whether its knockdown also caused presynapse pruning defects. To specifically visualize c4da neuron presynapses, we expressed a fluorescently tagged fragment of the presynaptic release site factor Bruchpilot (Brp), Brp^short^::Strawberry, under *ppk-GAL4*. In control animals at the larval stage, approximately 400 presynaptic Brp^short^::Strawberry puncta could be seen per segment in VNC segments A3-A5 (Fig. 6B,D), and UAP56 knockdown did not affect their number or distribution (Fig. 6C,D). At 24 h APF, c4da neuron axonal commissures had disappeared in control neurons, and the number of presynaptic Brp^short^::Strawberry puncta was strongly reduced to approximately 20 per segment (Fig. 6E,G). In UAP56 knockdown c4da neurons, axonal commissures were frequently still intact, and approximately 90 Brp^short^::Strawberry puncta could be detected per segment (Fig. 6F,G), indicative of presynapse pruning defects.

As loss of UAP56 affects expression of the actin-severing enzyme Mical, we investigated whether the presynapse pruning defects upon loss of UAP56 were accompanied by changes in presynaptic actin. To address this, we expressed the actin marker lifeact::GFP in c4da neurons and visualized it in the VNC. At 24 h APF, after axonal commissures were pruned, lifeact::GFP evenly labeled c4da neuron axons in control neurons (Fig. 6H). UAP56 knockdown led to persistence of c4da neuron axonal commissures at this time point, which were clearly labeled by lifeact::GFP (Fig. 6I). In addition, lifeact::GFP was also visible in blob-like structures in positions where synaptic sites reside (Fig. 6I, arrows). Thus, loss of UAP56 causes defects in presynapse pruning that could be linked to changes in actin remodeling.

### Presynapse pruning requires actin remodeling

The observation that loss of UAP56 causes lifeact::GFP accumulation at presynapses prompted us to test directly whether actin disassembly is required for c4da neuron presynapse pruning. To test for roles of actin disassembly and dynamics, we overexpressed the Actin G15S mutant, which increases the resistance of actin filaments against disassembly (Posern et al., 2004), and we knocked down the actin-severing enzymes Mical and cofilin [encoded by *twinstar* (tsr) in *Drosophila*]. Consistent with the idea that Mical disassembles actin filaments during dendrite pruning (Kirilly et al., 2009), both *Actin^G15S^* and Mical knockdown caused dendrite pruning defects at 18 h APF (Fig. S6). Despite its known role in larval c4da neuron dendrite morphogenesis (Nithianandam and Chien, 2018), cofilin was not required for dendrite pruning (Fig. S6).

**Fig. 7.**
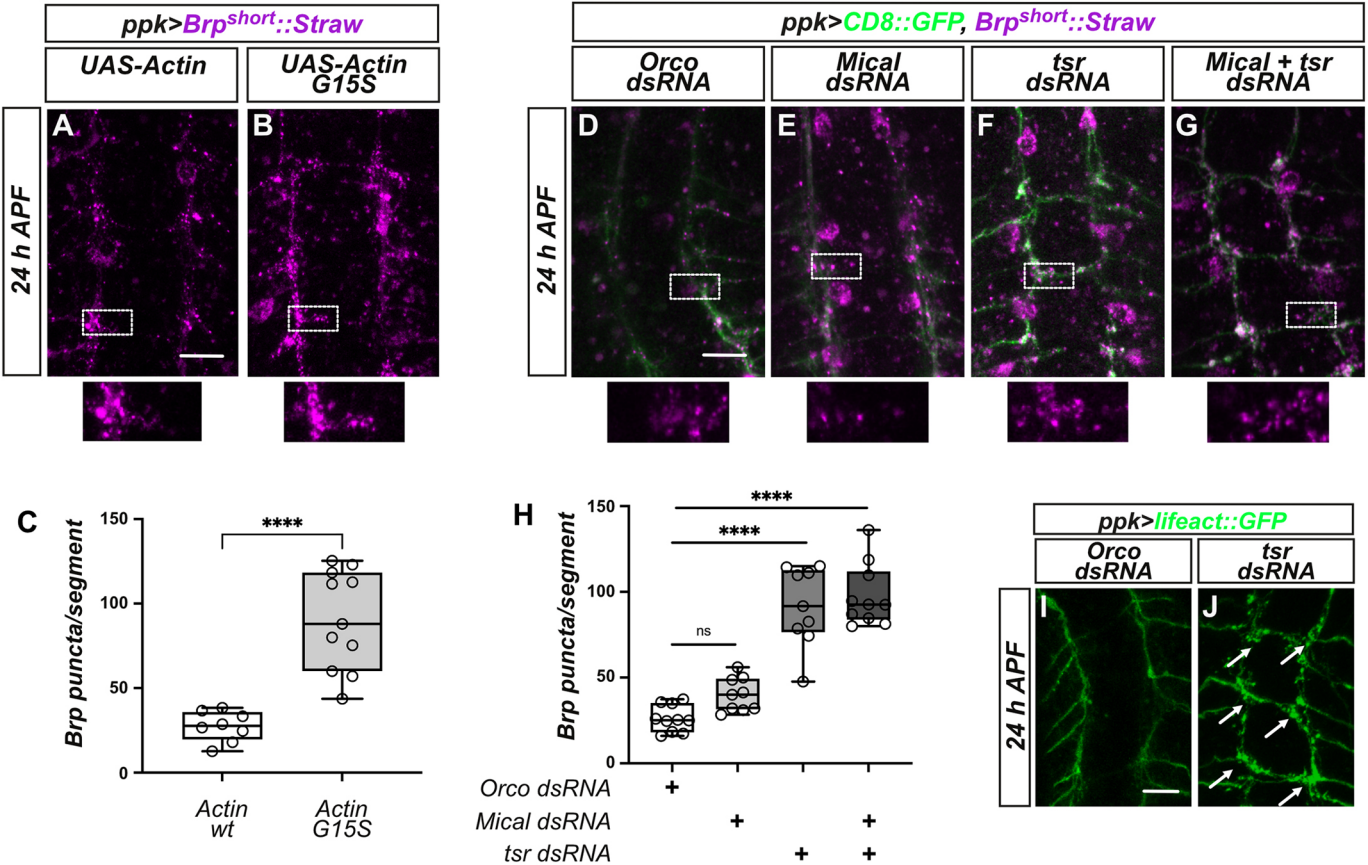
**Actin disassembly factors are required for c4da neuron presynapse pruning.** (A-C) Disassembly-resistant Actin G15S causes presynapse pruning defects. C4da neuron axons and presynapses in the VNC were labeled by expression of CD8::GFP and the active zone marker *Brp^short^::Strawberry* under the control of *ppk-GAL4*, and VNCs (segments A3-A5) were visualized at 24 h APF. Lower panels show magnified views of the *Brp^short^::Strawberry* signal in the boxed areas above. (A) Control c4da neurons expressing wild-type Actin. (B) C4da neurons expressing Actin G15S. (C) Quantification of the number of Brp puncta for the experiment shown in A,B. Analysis was carried out as described for Fig. 6. *n*=9-10 animals. ****P<0.0001 (unpaired *t*-test). (D-H) Actin disassembly factors are required for c4da neuron presynapse pruning. C4da neuron presynapses were analyzed at 24 h APF. Lower panels show magnified views of the *Brp^short^::Strawberry* signal in the boxed areas. (D) Control neurons expressing Orco dsRNA. (E) Neurons expressing *Mical* dsRNA. (F) Neurons expressing *tsr* dsRNA. (G) Neurons expressing both *Mical* and *tsr* dsRNA. (H) Quantification of the number of Brp puncta for the experiment shown in D-G. Analysis was carried out as described for Fig. 6. *n*=9-10 animals. ns, not significant. ****P<0.0001 (one-way ANOVA). (I,J) Loss of cofilin causes actin accumulation at c4da neuron presynapses during the pupal stage. F-actin in c4da neurons was labeled with *UAS-lifeact::GFP*, and c4da neuron presynaptic areas were visualized at 24 h APF. (I) Control c4da neurons expressing Orco dsRNA. (J) C4da neurons expressing *tsr* dsRNA. Actin accumulations are indicated by arrows. Scale bars: 10 µm (A,D,I).

At 24 h APF, over 80 Brp^short^::Strawberry puncta could be seen per segment in the presynapses of c4da neurons expressing *Actin^G15S^*, while there were only ∼25 Brp^short^::Strawberry puncta per segment when wild-type Actin was overexpressed (Fig. 7A-C), indicative of a role for actin disassembly during presynapse pruning. We therefore tested next whether actin disassembly factors are required for presynapse pruning. Mical knockdown increased the number of Brp puncta at 24 h APF only mildly and non-significantly compared to a control dsRNA (Fig. 7D,E,H). In contrast, cofilin knockdown caused strong synapse pruning defects (∼90 Brp puncta/segment) (Fig. 7F,H). Cofilin and Mical can act synergistically on bundled actin fibers (Rajan et al., 2023), but co-expression of Mical and cofilin dsRNAs did not significantly increase the number of remaining Brp^short^::Strawberry puncta compared to cofilin knockdown alone (Fig. 7G,H). Similar to UAP56 knockdown, *tsr* knockdown also led to persisting lifeact::GFP at commissures and in blob-like accumulations (Fig. 7I,J). Importantly, the observed defects were specific for presynapse pruning during metamorphosis, as combined Mical and *tsr* knockdown did not alter the number of presynaptic Brp^short^::Strawberry puncta at the larval stage (Fig. S7). Taken together, actin remodeling is broadly required for c4da neuron remodeling: while Mical is the principal actin disassembly factor during dendrite pruning, the actin disassembly factor during presynapse pruning is cofilin.

## DISCUSSION

In this work, we describe a role for the mRNA export factor UAP56 during c4da neuron neuronal remodeling. Using the advantages of the c4da neuron system, we investigated two mechanistic aspects of UAP56 involvement: the role of its ATPase activity and potential mechanisms during dendrite pruning.

Regarding the UAP56 ATPase, we investigated UAP56 mutations in the DECD motif that are known to inhibit the UAP56 ATPase. These mutants affected UAP56 localization and protein interactions, but rescued the pruning defects of a *uap56* mutant allele. It is known that several UAP56 molecules form part of each export intermediate mRNP, and that UAP56 ATPase is specifically required to remove Ref1, but not THO, from those complexes (Hohmann et al., 2025). One explanation for the observed functional rescue could be that removal of UAP56 and/or Ref1 is not stringently needed for nuclear export of mRNP particles. Since our mutant allele *uap56^k11511^* is a P element insertion in the 5'UTR and therefore likely a strong hypomorph, we cannot technically rule out the presence of residual endogenous UAP56 in the clones. The observed rescue could therefore also point to a mechanism of action whereby ATPase activity in only very few UAP56 molecules in an export complex may be required to support functional export. Importantly, UAP56 ATPase mutants did not have dominant-negative effects on dendrite pruning, which would be expected if coordinated ATP hydrolysis by all UAP56 molecules in an export complex was stringently needed.

UAP56 has also been shown to affect cytoplasmic mRNA localization in *Drosophila* oocytes (Meignin and Davis, 2008), opening up the possibility that nuclear mRNA export and cytoplasmic transport are linked. It remains to be seen whether this also applies to dendritic mRNA transport (Brechbiel and Gavis, 2008).

While a general inhibition of mRNA export might be expected to cause broad defects in many cellular processes, our data in developing c4da neurons indicate that loss of UAP56 mainly affects pruning mechanisms related to actin remodeling, but not microtubule regulation or the ubiquitin system. This could occur because some mRNA species are more sensitive to loss of UAP56 than others, or because there are other (redundant) mRNA export mechanisms. A candidate target of UAP56 during dendrite pruning is the actin-severing enzyme Mical, as both the amount of *Mical* mRNA in the cytoplasm and Mical protein expression are reduced upon loss of UAP56. In addition, loss of UAP56 also causes defects in presynapse pruning that are accompanied by accumulation of the actin marker lifeact::GFP at presynaptic sites. Indeed, we found that presynapse pruning also depends on actin disassembly, this time not by Mical, but by cofilin. While cofilin had previously been implicated in dendrite growth and branching (Nithianandam and Chien, 2018) and both pre- and postsynaptic processes such as negative regulation of neurotransmitter release and dendritic spine maturation (Rust, 2015), a role in synaptic pruning has not previously been described. Whether cofilin expression is directly affected by loss of UAP56 remains to be seen. Cofilin is known to be subject to extensive upstream regulation, e.g., by LIM kinases or Slingshot phosphatases (Rust, 2015), such that the effect of UAP56 on actin regulation at presynapses may not necessarily be directly via cofilin mRNA. Our data also reveal a specialization in actin remodeling factors: whereas Mical is the predominant actin-severing enzyme during c4da neuron dendrite pruning, cofilin is important during presynapse pruning. As Mical is more effective in disassembling bundled F-actin than cofilin (Rajan et al., 2023), it is interesting to speculate that such bundles are more prevalent in dendrites than at presynapses.

Taken together, our data provide new insights into the function of post-transcriptional gene regulation during neuronal development and reveal a role for actin remodeling during presynapse pruning.

## MATERIALS AND METHODS

### Fly strains

For expression in c4da neurons, we used *ppk-GAL4* insertions on the second and third chromosomes (Grueber et al., 2007) driving UAS-CD8::GFP. MARCM clones were induced with *SOP-FLP* (Matsubara et al., 2011) and labeled by UAS-tdtomato (Han et al., 2014) expression under *nsyb-GAL4^R57C10^* (Pfeiffer et al., 2008). Other UAS lines were UAS-EcR^DN^ [Bloomington *Drosophila* Stock Center (BL) 6872], UAS-MCP::RFP (BL 27418), UAS-VCP QQ (Rumpf et al., 2011), UAS-lifeact::GFP (BL 35544), UAS-Brp^short^::Strawberry (gift from S. Sigrist, Freie Universität Berlin, Germany), UAS-Actin5C G15S, UAS-Act42A wt (Hsiao et al., 2014), UAS-CD8::RFP (BL 27399). dsRNA lines against Orco (BL 31278) or mCherry (BL 35785) were used as controls, and UAP56 RNAi probes were VDRC (Vienna *Drosophila* Resource Center) 22557 (#1), BL 33666 (#2). Other dsRNA lines were against *tho2* (BL 28537), *thoc5* (BL 55206), *sbr* (#1: BL 28924; #2: BL34945), *Mical* (VDRC 20672 or NIGfly 18667R-2) and *tsr* (VDRC 110599). All dsRNAs were co-expressed with UAS-dcr2 (Dietzl et al., 2007) except in the *Mical*-MS2 experiment in Fig. 4. For MARCM, the P-element insertion *uap56^k11511^* (BL 11043) was recombined on FRT40A. Other fly lines were *ppk-EB1::GFP* (Arthur et al., 2015) and *act-Cas9, lig4^−/−^* (Zhang et al., 2014).

### Cloning and transgenes

C-terminally GFP-tagged UAP56 was cloned into pUAST attB, and the ATPase variants E194Q and E194A were generated by PCR mutagenesis. The corresponding plasmids were injected into flies harboring the 86Fb insertion site. The non-cleavable ubiquitin reporter Ub-VV-GFP was cloned into pUAST attB and introduced into flies harboring VK37 or VK16 insertion sites, and a recombinant with both insertions was used. In order to generate *Mical-MS2_6_* flies*,* we generated a specific sgRNA (AGTAGAGAGCCTAACTAATA) targeting the *Mical* 3′UTR in the pCFD3 vector. We then cloned tandem copies of the MS2 sequence [using pSL-MS2-6x (Addgene plasmid #27118) as a template] using the primers GGACGGATCCGCTTCTCCCATATTAGTTAGG (forward) and GGACCTCGAGAATGAACCCGGGAATACTG (reverse) and flanked them with 1.5 kb homology arms corresponding to sequences in the *Mical* CDS and 3′UTR. Primers for the flanking regions were: GGACGAATTCGTTTATGGCTTAAGTACTTGGC and GGACGGATCCGCTTCTCCCATATTAGTTAGG (left arm), and GGACCTCGAGGCGCAAGGTTAATGTAAAAACAG and GGACGCGGCCGCAAACACATCTCGTGACAACC (right arm). This homology donor was cloned into pBS SK(−), and the PAM motif for the sgRNA was mutated using PCR. The sgRNA and homology donor plasmids were then co-injected into *actin-Cas9, lig4^−/−^* embryos. Male injectants were crossed back, and male offspring was screened for the presence of MS2 by PCR. Positive lines were verified by PCR and sequencing, and two viable lines were chosen for experiments, both of which carried six copies of the MS2 sequences as assessed by sequencing. Ref1 and THOC7 coding sequences were cloned into pENTR and subsequently into the destination vectors pUAST-myc-rfa or pUAST-rfa-Dendra2 (Rumpf lab) using a TOPO cloning strategy.

### Dissection, microscopy and live imaging

For analyses of dendrite pruning phenotypes at 18 h APF, animals of either sex were dissected out of the pupal case and dorsal ddaC c4da neurons in segments A2-A5 were imaged live on a Zeiss LSM710 confocal microscope with a 20× Plan Apochromat water objective (1.0 NA). EB1::GFP imaging was performed on a Zeiss LSM880 microscope using a 40× Plan Apochromat FCS M27 (1.2 NA) oil objective. Consecutive images of a single plane were taken every second for 1-2 min. For analyses of presynapse pruning, pupae were dissected out of the pupal case at 24 h APF and dissected ventrally. The brain and VNC were removed and analyzed live using a Zeiss LSM710 confocal microscope with a 40× C-Apochromat water objective (1.1 NA). For reproducibility, only synapses in VNC segments A3-A5 were used for quantification. Ub-VV-GFP reporter fluorescence was assessed with a 40× C-Apochromat water objective (1.1 NA) at 3× zoom. For *Mical* mRNA analysis, UAS-MCP::RFP was expressed under *ppk-GAL4* in a *Mical-MS2_6_* background. Images of MCP::RFP and UAP56::GFP were taken live in undissected animals at the indicated developmental time points on LSM710 or LSM880 confocal microscopes using either a 40× C-Apochromat water objective (1.1 NA) or a 63× Plan-Apochromat oil objective (1.4 NA). All processing was performed in Fiji (Schindelin et al., 2012) using the plug-in Image Stabilizer for EB1::GFP comet analysis.

### Immunofluorescence

Antibodies against Mical (Rode et al., 2018) (1:500) and Sox14 (Ritter and Beckstead, 2010) (1:30) were used as described (Rumpf et al., 2014; Herzmann et al., 2017). The ubiquitin antibody was P4D1 (Santa Cruz Biotechnology, sc-8017) (1:50). Chicken anti-GFP antibodies (GFP-1020, Aves labs) (1:500) were used to visualize c4da neurons expressing CD8::GFP. Appropriately staged larvae and pupae were dissected, and body wall filets were fixed in 4% formaldehyde for 20 min, washed in PBS with 0.3% Triton X-100 and blocked in PBS with 0.3% Triton X-100 and 10% goat serum. Secondary antibodies were: goat anti-rabbit Alexa 568 (Invitrogen, A11011), goat anti-guinea pig Alexa 568 (Invitrogen, A11075), goat anti-mouse Alexa 568 (Invitrogen, A11004), goat anti-chicken Alexa 488 (Invitrogen, A11039). All antibody incubations were overnight.

### Immunoprecipitation and western blotting

pUAST expression plasmids encoding wild-type or mutant UAP56::GFP and myc-Ref1 were co-transfected into S2 cells with Actin5C-GAL4. After 72 h, cells were harvested in ice-cold PBS and lysed for 30 min in lysis buffer [100 mM NaCl, 5 mM Mg(OAc)_2_, 5% glycerol, 50 mM Tris-HCl pH 7.4, 1% Triton X-100, 1× complete protease inhibitors]. Cleared lysates were precipitated with mouse anti-Myc antibodies (9E10, in house hybridoma supernatant, gift from C. Klämbt, Universität Münster, Germany) bound to protein A sepharose for 2 h. After three washes in lysis buffer, precipitates were dissolved in SDS sample buffer at 95°C for 5 min, run on an 8% polyacrylamide gel and blotted with antibodies against Myc (9E10, 1:50), GFP (JL-8, Takara 632381, 1:1000) or THOC7 (Kim et al., 2011) (1:500). For the THOC7 experiment, lysates were precipitated with mouse anti-GFP antibodies (Invitrogen, A11222) bound to protein A sepharose for 2 h. Uncropped images of blots from this paper are shown in Fig. S8.

### Quantification and statistical analyses

Phenotypic penetrance was assessed by counting the number of neurons with dendrites still attached to the soma. Here, significance was determined using a categorical two-tailed Fisher's exact test (https://www.graphpad.com/). The number of primary and secondary dendrites attached to the soma (a measure of phenotypic strength) was counted manually and compared using the Wilcoxon Mann–Whitney test (Marx et al., 2016). Ub-VV-GFP fluorescence intensity was taken after background subtraction and samples were compared using a two-tailed Student's *t*-test. EB1 comet directionality across samples was compared using Fisher's exact test, and comet speed was compared with Wilcoxon's test using Prism software. The number of cytoplasmic MCP dots was counted manually, and compared using Student's *t*-test. To assess expression levels of Mical protein, Mical staining intensity in c4da neurons after background subtraction was normalized to its staining intensity in unaffected neighboring c1da neurons. Data were then compared using Wilcoxon's test. The number of synaptic Brp puncta per segment was determined from binarized confocal image stacks using the Fiji plugin SynQuant (Wang et al., 2020). Significance was determined in Prism using a *t*-test, Mann–Whitney test or one-way ANOVA, depending on normal distribution and number of samples.

## Supplementary Material



10.1242/joces.264770_sup1 Supplementary information
