# Supplementary material for: The mRNA export factor UAP56 is required for dendrite and synapse pruning via actin regulation in Drosophila
Source: J Cell Sci. 2026 May 11;139(9):jcs264770. doi: 10.1242/jcs.264770 (PMC13245899; doi:10.1242/jcs.264770)
Supplement: Supplementary information [file joces-139-264770-s1.pdf]

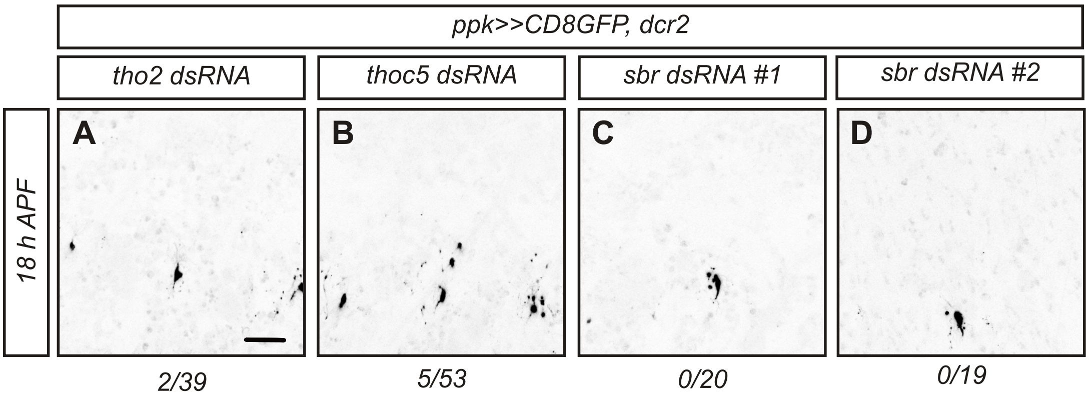

Fig. S1. Knockdown of mRNA export factors in c4da neurons and effect on dendrite pruning. RNAi constructs targeting the indicated export factors were expressed under ppk-GAL4, and c4da neuron morphology was assessed at 18 h APF. Numbers below panels indicate neurons with pruning defects versus total number of neurons assessed. The scale bar in **A** is 50 μm.

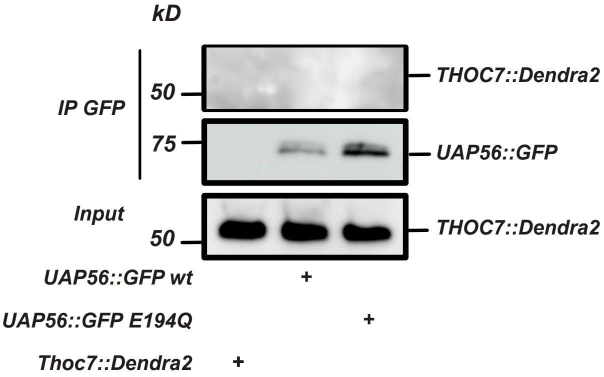

Fig. S2. Co-immunoprecipitation between UAP56::GFP wt or UAP56::GFP E194Q and THOC7. GFP-tagged UAP56 constructs (**B**: wt and E194A, **C** wt and E194Q) were coexpressed with THOC7::Dendra2 in S2 cells, and precipitated using anti-GFP antibodies. Shown are bound fractions (top panels) blotted against GFP and THOC7, and input fractions blotted for THOC7 (bottom). The position of a molecular weight marker is indicated to the left.

Journal of Cell Science • Supplementary information

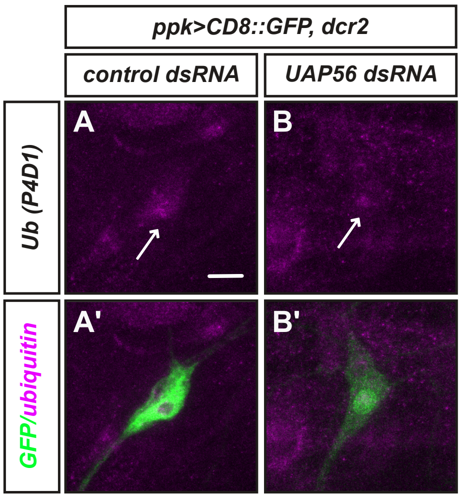

Fig. S3. Effect of UAP56 loss on ubiquitin levels in c4da neurons. C4da neurons were labeled by *CD8::GFP* under the control of *ppk-GAL4* and ubiquitin was detected by immunofluorescence at the third instar. **A**, **A'** Control c4da neuron expressing Orco dsRNA. **B**, **B'** C4da neuron expressing UAP56 dsRNA. The scale bar in **A** is 10 µm.

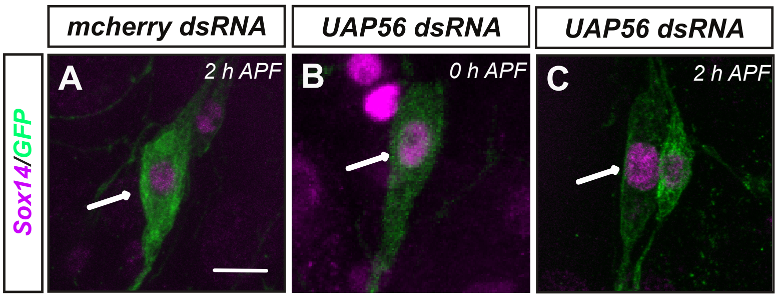

Fig. S4. Sox14 expression in c4da neurons lacking UAP56. C4da neurons were labeled by *CD8::GFP* under the control of *ppk-GAL4* and Sox14 was detected by immunofluorescence. **A** Control c4da neuron expressing mcherry dsRNA at 2 h APF. **B** C4da neuron expressing UAP56 dsRNA at 0 h APF. **C** C4da neuron expressing UAP56 dsRNA at 2 h APF. The scale bar in **A** is 10 µm.

Journal of Cell Science • Supplementary information

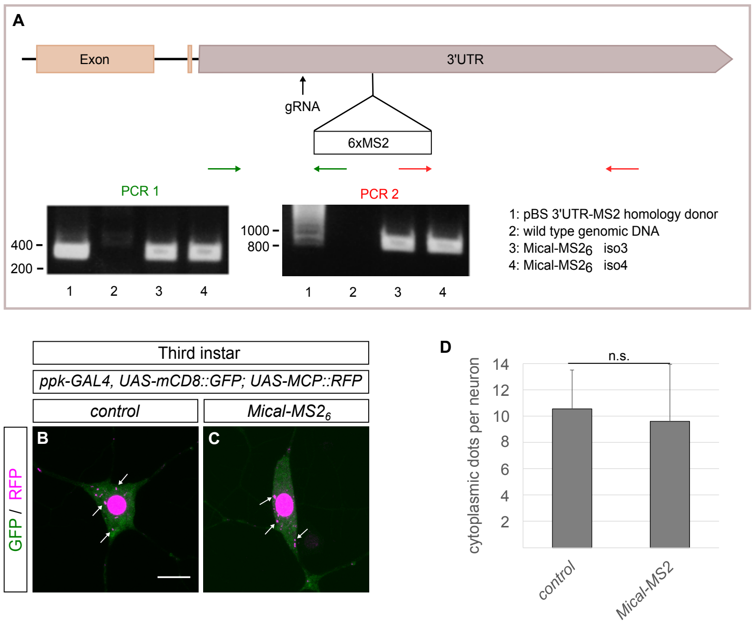

Fig. S5. Generation and characterization of *Mical-MS2₆*. **A** Strategy for generation of *Mical-MS2₆* flies. The schematic shows the positions of the sgRNA target relative to the MS2 sequence insertion site and those of the PCR primers used to confirm successful insertion. The agarose gels show PCR confirmation of two positive fly lines with the primers indicated above. Running positions of size markers are indicated on the left of each gel. **B**, **C** Images of third instar larval c4da neuron somata expressing MCP::RFP under the control of *ppk-GAL4*. **B** C4da neuron expressing MCP::RFP in wild type background. **C** C4da neuron expressing MCP::RFP in *Mical-MS2₆* background. **D** Quantification of the number of cytoplasmic MCP::RFP puncta in **B**, **C**. N=10 each, n. s., not significant, Student's t-test. The scale bar in **B** is 10 μm.

Journal of Cell Science • Supplementary information

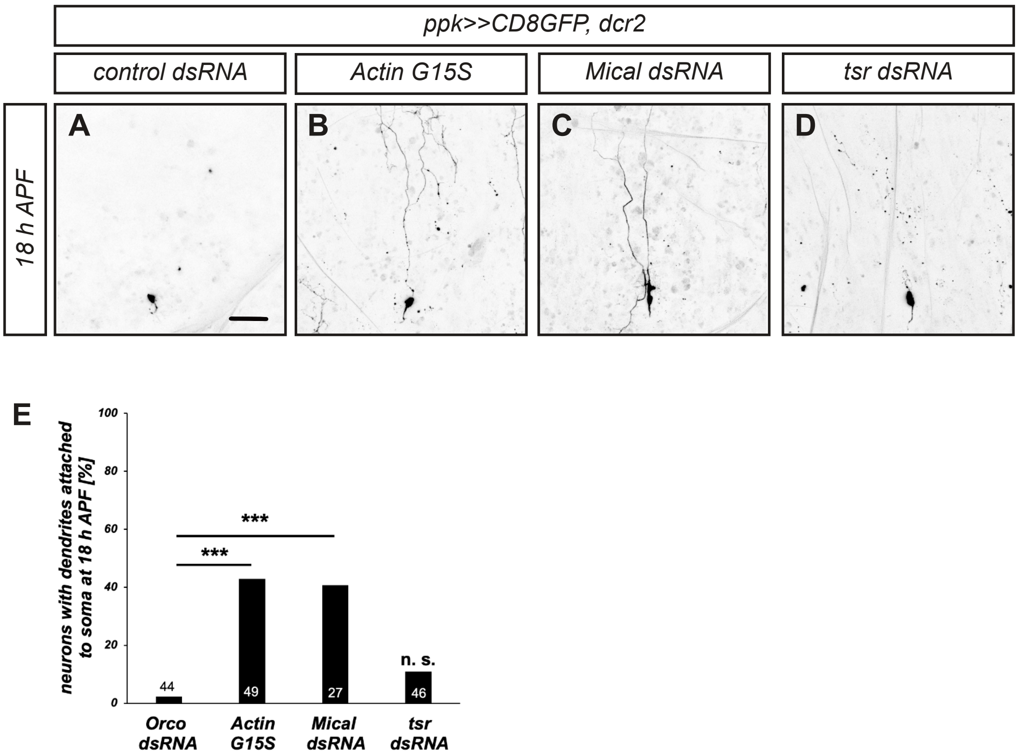

Fig. S6. Actin manipulations and c4da neuron dendrite pruning. A - D RNAi constructs targeting the actin severing factors Mical (C) or cofilin (*twinstar/tsr*) (D) or the disassembly-resistant actin mutant Actin G15S (B) were expressed under ppk-GAL4, and c4da neuron morphology was assessed at 18 h APF. Orco dsRNA served as control (A). **E** Quantification of penetrance of dendrite pruning defects in A - D. N=27 - 49, *** P<0.0005, Fisher's exact test. The scale bar in **A** is 50 µm.

Journal of Cell Science • Supplementary information

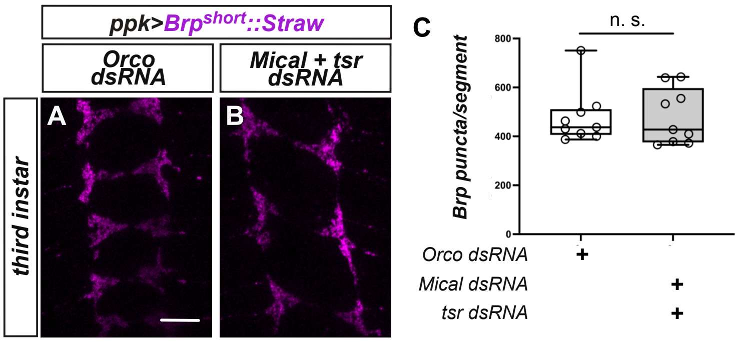

Fig. S7. Loss of Actin disassembly factors does not affect the number of presynaptic sites at the third instar. Active zones labeled by *Brp^{short}::Strawberry* were visualized at the third instar stage. **A** C4da neuron active zones in control larvae expressing Orco dsRNA. **B** C4da neuron active zones at 24 h APF in larvae expressing both Mical and tsr dsRNA. **C** Quantification of the number of Brp puncta in **A**, **B**. N=9 each, n. s., not significant, Mann-Whitney U-test. Scale bar in **A** is 10 μm.

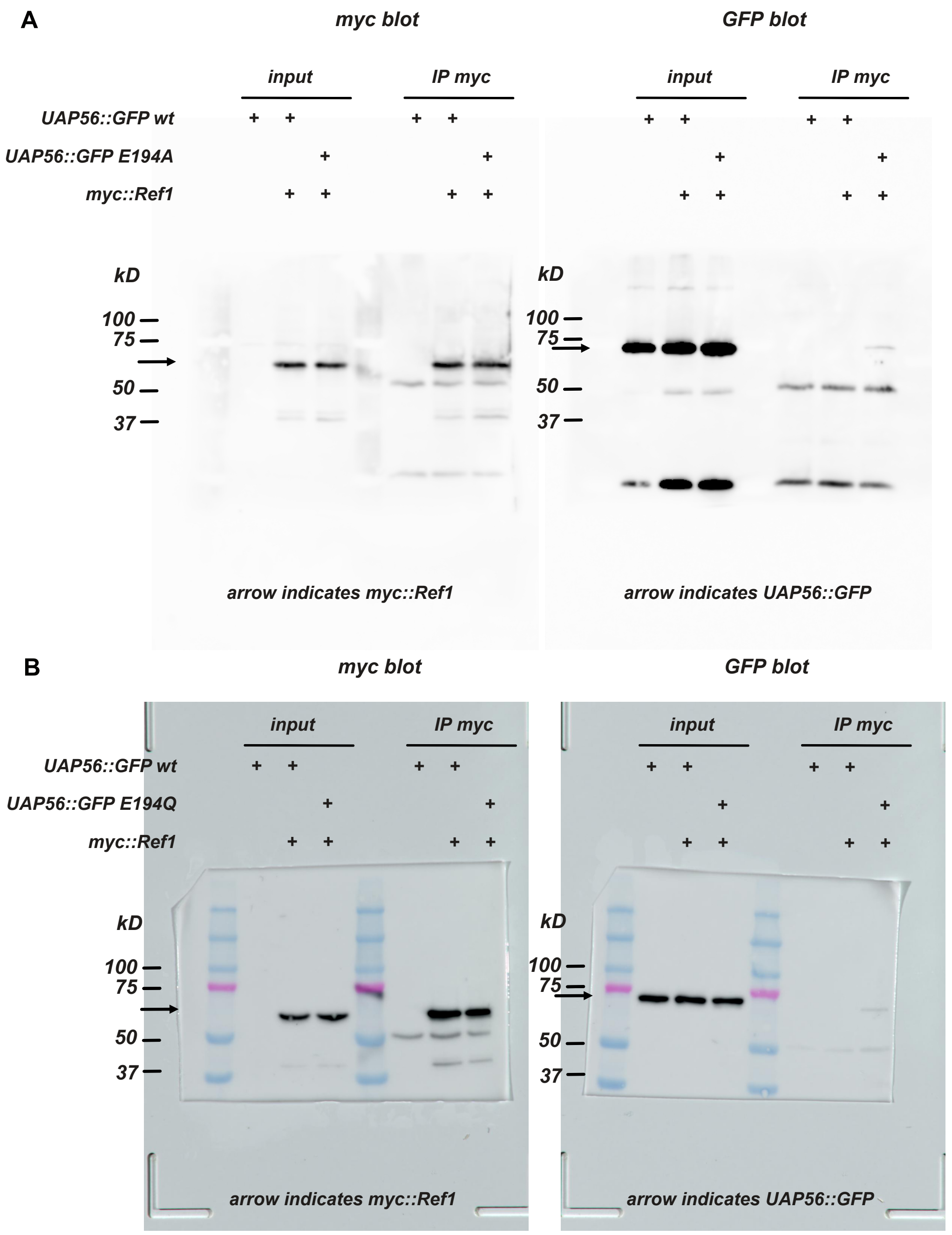

Fig. S8. Blot transparency. Shown are the uncropped Western blots from Figures 2B, 2C and Figure S2. Positions of molecular weight markers is shown. **A** Original blots for Fig. 2 B (interaction between UAP56::GFP EA and myc::Ref1, anti-myc and anti-GFP blots). **B** Original blots for Fig. 2 C (interaction between UAP56::GFP EQ and myc::Ref1, anti-myc and anti-GFP blots).

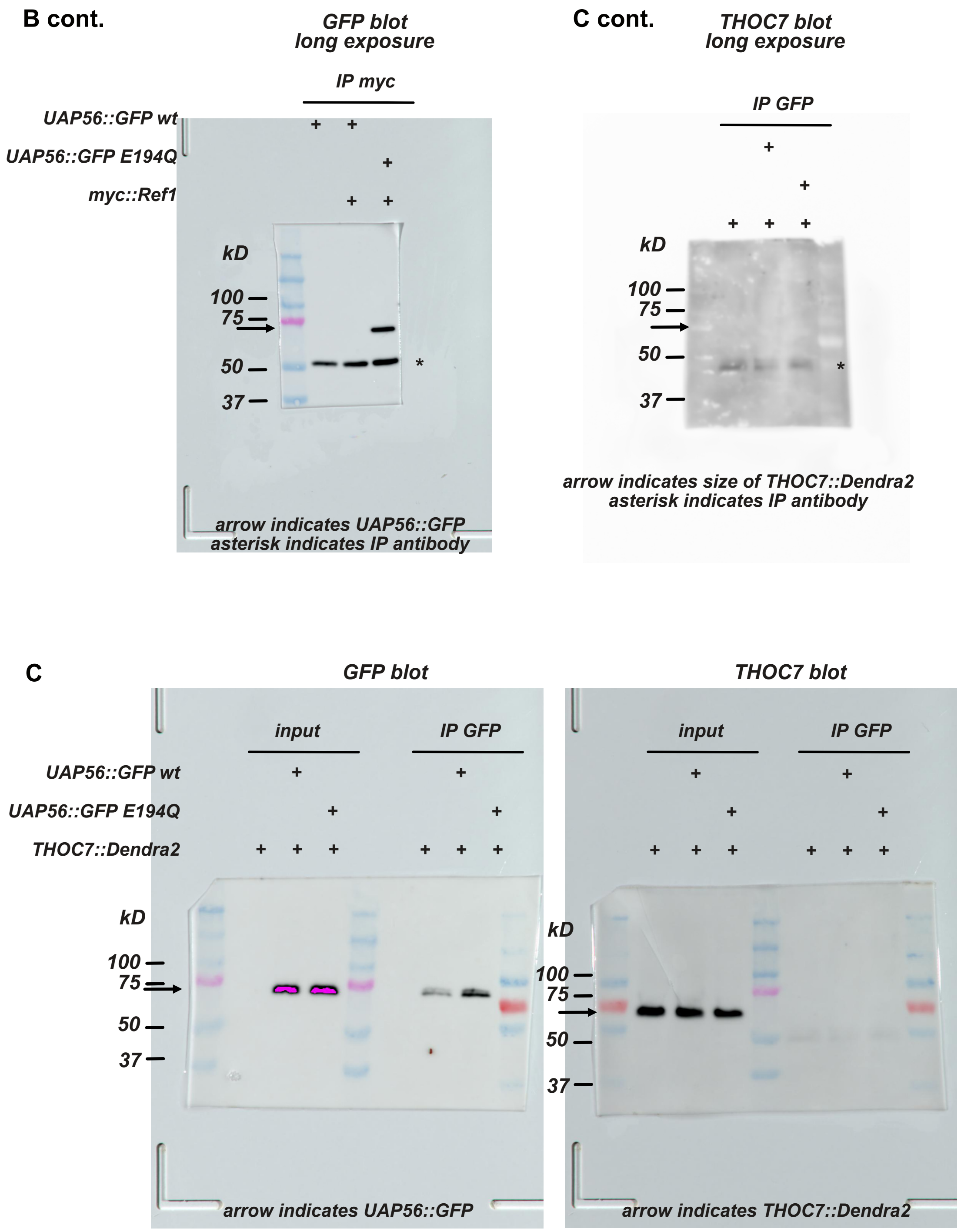

Fig. S8 continued. Blot transparency. B cont. Long exposure of the anti-GFP blot of the myc IP samples. **C** Original blots for Fig. S2 (no detectable interaction between UAP56::GFP and THOC7, anti-GFP and anti-THOC7 blots; top: long exposure of THOC7 blot of IP samples).

Journal of Cell Science • Supplementary information

Table S1. Screen for RNA-binding proteins required for c4da neuron dendrite pruning. 103 RNAi lines directed against 64 different genes were analyzed for pruning defects at 18 h APF upon expression in c4da neuron under the control of *ppk-GAL4*. Lines where more than 10 percent of the analyzed neurons retained attached dendrites at 18 h APF were scored as hits. N > 10 neurons each.

Symbol	CG	Transforment ID	Pruning defect
aret	CG31762	41567 (VDRC)	no
aret	CG31762	41568 (VDRC)	no
aret	CG31762	48237 (VDRC)	no
B52	CG10851	38860 (VDRC)	no
B52	CG10851	38862 (VDRC)	no
bl	CG13425	2912 (VDRC)	no
Brr2	CG5931	43962 (VDRC)	no
Caz	CG3606	100291 (VDRC)	no
Cbp20	CG12357	50433 (VDRC)	no
cdc2rk	CG1362	32249 (VDRC)	no
CG10418	CG10418	50245 (VDRC)	no
CG10445	CG10445	104753 (VDRC)	no
CG11360	CG11360	38491 (VDRC)	no
CG11360	CG11360	38492 (VDRC)	no
CG14641	CG14641	38790 (VDRC)	no
CG1646	CG1646	32682 (VDRC)	no
CG1646	CG1646	32683 (VDRC)	no
CG16941	CG16941	20338 (VDRC)	no
CG17454	CG17454	20458 (VDRC)	no
CG2926	CG2926	33589 (VDRC)	no
CG2926	CG2926	33591 (VDRC)	no
CG4119	CG4119	26395 (VDRC)	no
CG4119	CG4119	106696 (VDRC)	no
CG5728	CG5728	24696 (VDRC)	no
CG5728	CG5728	24697 (VDRC)	no
CG6227	CG6227	40351 (VDRC)	no
CG6227	CG6227	40352 (VDRC)	no
CG6841	CG6841	34253 (VDRC)	no

CG6841	CG6841	34254 (VDRC)	no
CG6999	CG6999	41828 (VDRC)	no
CG7185	CG7185	107147 (VDRC)	no
CG7879	CG7879	15260 (VDRC)	no
crn	CG3193	28815 (VDRC)	no
crn	CG3193	28816 (VDRC)	no
crn	CG3193	39335 (VDRC)	no
cyp33	CG4886	108734 (VDRC)	no
Cypl	CG13892	44823 (VDRC)	no
Dbp45A	CG12759	17306 (VDRC)	no
Doa	CG42320	19066 (VDRC)	no
Doa	CG42320	46449 (VDRC)	no
dom	CG9696	7787 (VDRC)	no
dom	CG9696	7789(VDRC)	no
eIF4A	CG9075	42201 (VDRC)	**yes**
eIF4A	CG9075	42202 (VDRC)	**yes**
eIF4A	CG9075	32970 (BI)	**yes**
eIF4AIII	CG7483	108580 (VDRC)	no
eIF4AIII	CG7483	32444 (BL)	no
eIF4AIII	CG7483	32907 (BL)	no
fand	CG6197	46312 (VDRC)	no
hay	CG8019	41022 (VDRC)	no
Hel25E	CG7269	22556 (VDRC)	**yes**
Hel25E	CG7269	22557 (VDRC)	**yes**
Hel25E	CG7269	33666 (BI)	**yes**
how	CG10293	100775 (VDRC)	no
hrp36	CG12749	100732 (VDRC)	no
Lsm11	CG12924	20280 (VDRC)	no
mago	CG9401	28132 (VDRC)	no
Mfap1	CG1017	15610 (VDRC)	no
Mfap1	CG1017	103419 (VDRC)	no
mub	CG7437	28024 (VDRC)	no
mub	CG7437	105495 (VDRC)	no
pea	CG8241	47782 (VDRC)	no
Prp18	CG6011	13760 (VDRC)	no
Prp19	CG5519	22146 (VDRC)	no
Prp19	CG5519	22147 (VDRC)	no
Prp19	CG5519	41438 (VDRC)	no
Prp22	CG8241	36558 (VDRC)	no
Prp3	CG7757	25547 (VDRC)	no

Journal of Cell Science • Supplementary information

Prp3	CG7757	25548 (VDRC)	no
Prp31	CG6876	35131 (VDRC)	no
Prp38	CG30342	21136 (VDRC)	no
Prp8	CG8877	18565 (VDRC)	no
Prp8	CG8877	18567 (VDRC)	no
ps	CG8144	24214 (VDRC)	no
pUf68	CG12085	20144 (VDRC)	no
RM62	CG10279	46908 (VDRC)	no
RM62	CG10279	110102 (VDRC)	no
RM62	CG10279	31395 (BI)	no
RM62	CG10279	34829 (BI)	no
Rox8	CG5422	27415 (VDRC)	no
Rox8	CG5422	28649 (VDRC)	no
Rox8	CG5422	41439 (VDRC)	no
SC35	CG5442	104978 (VDRC)	no
snf	CG4528	104334 (VDRC)	no
Spf45	CG17540	32948 (VDRC)	no
Spf45	CG17540	32949 (VDRC)	no
sqd	CG16901	32395 (VDRC)	no
Srp54	CG4602	51088 (VDRC)	no
Sxl	CG43770	3132 (VDRC)	no
Sxl	CG43770	50786 (VDRC)	no
tst	CG10210	38356 (VDRC)	no
tsu	CG8781	36024 (VDRC)	no
tsu	CG8781	107385 (VDRC)	no
tsu	CG8781	28955 (BI)	no
U1-70K	CG8749	23150 (VDRC)	no
U1-70K	CG8749	23151 (VDRC)	no
U1C	CG5454	22132 (VDRC)	no
U1C	CG5454	22133 (VDRC)	no
U2A	CG1406	17358 (VDRC)	no
U2A	CG1406	109815 (VDRC)	no
U2af38	CG3582	110075 (VDRC)	no
U2af50	CG9998	24176 (VDRC)	no
U2af50	CG9998	24177 (VDRC)	no

Journal of Cell Science • Supplementary information
