# Supplementary material for: The mRNA export factor UAP56 is required for dendrite and synapse pruning via actin regulation in Drosophila
Source: J Cell Sci. 2026 May 11;139(9):jcs264770. doi: 10.1242/jcs.264770 (PMC13245899; doi:10.1242/jcs.264770)
Supplement: Supplementary file 1 [file joces-139-264770_review_history.pdf]

The mRNA export factor UAP56 is required for dendrite and synapse pruning via actin regulation in *Drosophila*

Samuel Matthew Frommeyer, Ulrike Gigengack, Sandra Rode, Matthew Davies, Neele Wolterhoff and Sebastian Rumpf
DOI: 10.1242/jcs.264770

Editor: Subhojit Roy

Review timeline

Submission to Review Commons:	29 July 2025
Submission to Journal of Cell Science:	4 February 2026
Accepted:	24 March 2026

Reviewer 1

Evidence, reproducibility and clarity

Summary:

This manuscript by Frommeyer et al. explores the role of the helicase and regulator of nuclear export, UAP56, in the control of dendrite and presynaptic pruning in Drosophila larval da sensory neurons. The authors present evidence showing that UAP56 regulates these processes via the actin cytoskeleton and suggest that this is occurs by controlling the expression of the actin severing enzyme, Mical.

Major comments:

The most signficant issue with the manuscript is that some of the major conclusions are not supported by the data. Additional experiment would need to be completed in order support these claims. These (and other) major comments are as follows:

1. For Figure 4, the ms2/MCP system is not quantitative. Using this technique, it is impossible to determine how many RNAs are located in each "dot". Each of these dots looks quite large and likely corresponds to some phase-separated RNP complex where multiple RNAs are stored and/or transported. Thus, these data do not support the conclusion that Mical mRNA levels are reduced upon UAP56 knockdown. A good quantitative microscopic assay would be something like smFISH. Additinally, the localization of Mical mRNA dots to dendrites is not convincing as it looks like regions where there are dendritic swellings, the background is generally brighter.

2. Alternatively, levels of Mical mRNA could be verified by qPCR in the laval brain following pan-neuronal UAP56 knockdown or in FACS-sorted fluorescently labeled da sensory neurons. Protein levels could be analyzed using a similar approach.

3. In Figure 5, the authors state that Mical expression could not be detected at 0 h APF. The data presented in Fig. 5C, D suggest the opposite as there clearly is some expression. Moreover, the data shown in Fig. 5D looks significantly brighter than the Orco dsRNA control and appears to localize to some type of cytoplasmic granule. So the expression of Mical does not look normal.

4. Sufficient data are not presented to conclude any specificity in mRNA export pathways. Data is presented for one export protein (UAP56) and one putative target (Mical). To adequately assess this, the authors would need to do RNA-seq in UAP56 mutants.

© 2026. Published by The Company of Biologists under the terms of the Creative Commons Attribution License (https://creativecommons.org/licenses/by/4.0/).

5. In summary, better quantitative assays should be used in Figures 4 and 5 in order to conclude the expression levels of either mRNA or protein. In its current form, this study demonstrates the novel finding that UAP56 regulates dendrite and presynaptic pruning, potentially via regulation of the actin cytoskeleton. However, these data do not convincingly demonstrate that UAP56 controls these processes by regulating of Mical expression and defintately not by controlling export from the nucleus.

6. While there are clearly dendrites shown in Fig. 1C', the cell body is not readily identifiable. This makes it difficult to assess attachment and suggests that the neuron may be dying. This should be replaced with an image that shows the soma.

7. The level of knockdown in the UAS56 RNAi and P element insertion lines should be determined. It would be useful to mention the nature of the RNAi lines (long/short hairpin). Some must be long since Dcr has been co-expressed. Another issue raised by this is the potential for off-target effects. shRNAi lines would be preferable because these effects are minimized.

Minor comments:

1. The authors should explain what EB1:GFP is marking when introduced in the text.

2. The da neuron images througout the figures could be a bit larger.

Significance

Strengths:

The methodology used to assess dendrite and presynaptic prunings are strong and the phenotypic analysis is conclusive.

Weakness:

The evidence demonstrating that UAP56 regulates the expression of Mical is unconvincing. Similarly, no data is presented to show that there is any specificity in mRNA export pathways. Thus, these major conclusions are not adequately supported by the data.

Advance:

The findings that UAP56 regulate dendrite and synaptic pruning are novel. As is its specific regulation of the actin cytoskeleton. These findings are restricted to a phenotypic analysis and do not show that it is not simply due to the disruption of general mRNA export.

Audience:

In its current form the manuscript whould be of interest to an audience who specializes in the study of RNA binding proteins in the control of neurodevelopment. This would include scientists who work in invertebrate and vertebrate model systems.

My expertise:

My lab uses Drosophila to study the role of RNA binding proteins in neurodevelopment and neurodegeneration. Currently, we use flies as a model to better understand the molecular pathogenesis of neurodevelopmenal disorders such as FXS and ASD.

Reviewer 2

Evidence, reproducibility and clarity

In this paper, the authors describe dendrite pruning defects in c4da neurons in the DEXD box ATPase UAP56 mutant or in neuronal RNAi knockdown. Overexpression UAP56::GFP or

© 2026. Published by The Company of Biologists under the terms of the Creative Commons Attribution License (https://creativecommons.org/licenses/by/4.0/).

UAP56::GFPE194Q without ATPase activity can rescue dendrite pruning defects in UAP56 mutant. They further characterized the mis-localization of UAP56::GFPE194Q and its binding to nuclear export complexes. Both microtubules and the Ubiquitin-proteasome system are intact in UAP56RNAi neurons. However, they suggest a specific effect on MICAL mRNA nuclear export shown by using the MS2-MCP system., resulting in delay of MICAL protein expression in pruned neurons. Furthermore, the authors show that UAP56 is also involved in presynaptic pruning of c4da neuros in VNC and Mica and actin are also required for actin disassembly in presynapses. They propose that UAP56 is required for dendrite and synapse pruning through actin regulation in Drosophila. Following are my comments.

Major comments

1. The result that UAP56::GFPE194Q rescues the mutant phenotype while the protein is largely mis-localized suggests a novel mechanism or as the authors suggested rescue from combination of residual activities. The latter possibility requires further support, which is important to support the role mRNA export in dendrite and pre-synapse pruning. One approach would be to examine whether other export components like REF1, and NXF1 show similar mutant phenotypes. Alternatively, depleting residual activity like using null mutant alleles or combining more copies of RNAi transgenes could help.

2. The localization of UAP56::GFP (and E194Q) should be analyzed in more details. It is not clear whether the images in Fig. 2A and 2B are from confocal single sections or merged multiple sections. The localization to the nuclear periphery of UAP56::GFP is not clear, and the existence of the E194Q derivative in both nucleus and cytosol (or whether there is still some peripheral enrichment) is not clear if the images are stacked.

3. The Ub-VV-GFP is a new reagent, and its use to detect active proteasomal degradation is by the lack of GFP signals, which could be also due to the lack of expression. The use of Ub- QQ-GFP cannot confirm the expression of Ub-VV-GFP. The proteasomal subunit RPN7 has been shown to be a prominent component in the dendrite pruning pathway (Development 149, dev200536). Immunostaining using RPN7 antibodies to measure the RPN expression level could be a direct way to address the issue whether the proteasomal pathway is affected or not.

4. Using the MS2/MCP system to detect the export of MICAL mRNA is a nice approach to confirm the UAP56 activity; lack of UAP56 by RNAi knockdown delays the nuclear export of MS2-MICAL mRNA. The rescue experiment by UAS transgenes could not be performed due to the UAS gene dosage, as suggested by the authors. However, this MS2-MICAL system is also a good assay for the requirement of UAP56 ATPase activity (absence in the E194Q mutant) in this process. Could authors use the MARCM (thus reduce the use of UAS-RNAi transgene) for the rescue experiment? Also, the c4da neuronal marker UAS-CD8-GFP used in Fig4 could be replaced by marker gene directly fused to ppk promoter, which can save a copy of UAS transgene. The results from the rescue experiment would test the dependence of ATPase activity in nuclear export of MICAL mRNA.

5. The UAP56 is also involved in presynaptic pruning through regulating actin assembly, and the authors suggest that Mical and cofilin are involved in the process. However, direct observation of lifeact::GFP in Mical or cofilin RNAi knockdown is important to support this conclusion.

Minor comments

1. RNA localization is important for dendrite development in larval stages (Brechbiel JL, Gavis ER. Curr Biol. 20;18(10):745-750). Yet, the role of UAP56 is relatively specific and shown only in later-stage pruning. It would need thorough discussion.

2. Could authors elaborate on the possible upstream regulators that might be involved, as described in "alternatively, several cofilin upstream regulators have been described (Rust, 2015) which might also be involved in presynapse pruning and subject to UAP56 regulation" in Discussion?

3. In Discussion, the role of cofilin in pre- and post-synaptic processes was described. The role

© 2026. Published by The Company of Biologists under the terms of the Creative Commons Attribution License (https://creativecommons.org/licenses/by/4.0/).

of Tsr/Cofilin regulating actin behaviors in dendrite branching has been described in c3da and c4da neurons (Nithianandam and Chien, 2018 and other references) should be included in Discussion.

4. The authors speculate distinct actin structures have to be disassembled in dendrite and presynapse pruning in Discussion. What are the possible actin structures in both sites could be elaborated.

Significance

The study initiated a genetic screen for factors involved in a dendrite pruning system and reveals the involvement of nuclear mRNA export is an important event in this process. They further identified the mRNA of the actin disassembly factor MICAL is a candidate substrate in the exporting process. This is consistent with previous finding that MICAL has to be transcribed and translated when pruning is initiated. As the presynapses of the model c4da neuron in this study is also pruned, the dependence on nuclear export and local actin remodeling were also shown. Thus, this study has added another layer of regulation (the nuclear mRNA export) in c4da neuronal pruning, which would be important for the audience interested in neuronal pruning. The study is limited for the confusing result whether ATPase activity of the exporting factor is required.

Reviewer 3

Evidence, reproducibility and clarity

Summary:

In the manuscript by Frommeyer, Gigengack et al. entitled "The UAP56 mRNA Export Factor is Required for Dendrite and Synapse Pruning via Actin Regulation in Drosophila" the authors surveyed a number of RNA export/processing factors to identify any required for efficient dendrite and/or synapse pruning. They describe a requirement for a general poly(A) RNA export factor, UAP56, which functions as an RNA helicase. They also study links to aspects of actin regulation.

Overall, while the results are interesting and the impact of loss of UAP56 on the pruning is intriguing, some of the data are overinterpreted as presented. The argument that UAP56 may be specific for the MICAL RNA is not sufficiently supported by the data presented. The two stories about poly(A) RNA export/processing and the actin regulation seem to not quite be connected by the data presented. The events are rather distal within the cell, making connecting the nuclear events with RNA to events at the dendrites/synapse challenging.

There are a number of specific statements that are not supported by references. See, for example, these sentences within the Introduction- "Dysregulation of pruning pathways has been linked to various neurological disorders such as autism spectrum disorders and schizophrenia. The cell biological mechanisms underlying pruning can be studied in Drosophila." The Drosophila sentence is followed by some specific examples that do include references. The authors also provide no reference to support the variant that they create in UAP56 (E194Q) and whether this is a previously characterized fly variant or based on an orthologous protein in a different system. If so, has the surprising mis-localization been reported in another system?

Specific Comments:

Figure 1 shows the impact of loss of UAP56 on neuron dendrite pruning. The experiment employs both two distinct dsRNAs and a MARCM clone, providing confidence that there is a defect in pruning upon loss of UAP56. As the authors mention screening against 92 genes that caused splicing defects in S2 cells, inclusion of some examples of these genes that do not show such a defect would enhance the argument for specificity with regard to the role of UAP56. This control would be in addition to the more technical control that is shown, the mCherry dsRNA. Later the authors demonstrate a delay in the accumulation of the Mical protein, so if they assayed these pruning events at later times, would the loss of UAP56 cause a delay in these events as well? Such

© 2026. Published by The Company of Biologists under the terms of the Creative Commons Attribution License (https://creativecommons.org/licenses/by/4.0/).

a correlation would enhance the causality argument the authors make for Mical levels and these pruning events.

Figure 2 provides data designed to test the requirement for the ATPase/helicase activity of UAP56 for these trimming events. The first observation, which is surprising, is the mislocalization of the variant (E194Q) that the authors generate. The data shown does not seem to indicate how many cells the results shown represent as a single image and trace is shown the UAP56::GFP wildtype control and the E194Q variant.

Given the rather surprising finding that the ATPase activity is not required for the function of UAP56 characterized here, the authors do not provide sufficient references or rationale to support the ATPase mutant that they generate. The E194Q likely lies in the Walker B motif and is equivalent to human E218Q, which can prevent proper ATP hydrolysis in the yeast Sub2 protein. There is no reference to support the nature of the variant created here.

Given the surprising results, the authors could have included additional variants to ensure the change has the biochemical effect that the authors claim. Previous studies have defined missense mutations in the ATP-binding site- K129A (Lysine to Alanine): This mutation, in both yeast Sub2 and human UAP56, targets a conserved lysine residue that is critical for ATP binding. This prevents proper ATP binding and consequently impairs helicase function. There are also missense mutations in the DEAD-box motif, (Asp-Glu-Ala-Asp) involved in ATP binding and hydrolysis. Mutations in this motif, such as D287A in yeast Sub2 (corresponding to D290A in human UAP56), can severely disrupt ATP hydrolysis, impairing helicase activity. In addition, mutations in the Walker A (GXXXXGKT) and Walker B motifs are can impair ATP binding and hydrolysis in DEAD-box helicases. Missense mutations in these motifs, like G137A (in the Walker A motif), can block ATP binding, while E218Q (in the Walker B motif)- which seems to be the basis for the variant employed here- can prevent proper ATP hydrolysis.

The co-IP results shown in Figure 2C would also seem to have multiple potential interpretations beyond what the authors suggest, an inability to disassemble a complex. The change in protein localization with the E194Q variant could impact the interacting proteins. There is no negative control to show that the UAP56-E194Q variant is not just associated with many, many proteins. Another myc-tagged protein that does not interact would be an ideal control.

With regard to *Figure 3*, the authors never define EB1::GFP in the text of the Results, so a reader unfamiliar with this system has no idea what they are seeing. Reading the Materials and Methods does not mitigate this concern as there is only a brief reference to a fly line and how the EB1::GFP is visualized by microscopy. This makes interpretation of the data presented in Figure 3A-C very challenging.
The data shown for MICAL MS2 reporter localization in Figure 4 is nice, but is also fully expected on many former studies analyzing loss of UAP56 or UAP56 hypomorphs in different systems. While creating the reporter is admirable, to make the argument that MICAL localization is in some way preferentially impacted by loss of UAP56, the authors would need to examine several other transcripts. As presented, the authors can merely state that UAP56 seems to be required for the efficient export of an mRNA transcript, which is predicted based on dozens of previous studies dating back to the early 2000s.

Minor (and really minor) points:

In the second sentence of the Discussion, the word 'developing' seems to be mis-typed "While a general inhibition of mRNA export might be expected to cause broad defects in cellular processes, our data in develoing c4da neurons indicate that loss of UAP56 mainly affects pruning mechanisms related to actin remodeling."

Sentence in the Results (lack of page numbers makes indicating where exactly a bit tricky)- "We therefore reasoned that Mical expression could be more challenging to c4da neurons." This is a complete sentence as presented, yet, if something is 'more something'- the thing must be 'more than' something else. Presumably, the authors mean that the length of the MICAL transcript could make the processing and export of this transcript more challenging than typical fly transcripts (raising the question of the average length of a mature transcript in flies?).

© 2026. Published by The Company of Biologists under the terms of the Creative Commons Attribution License (https://creativecommons.org/licenses/by/4.0/).

Significance

Understanding how post-transcriptional events are linked to key functions in neurons is important and would be of interest to a broad audience.

Author response to reviewers' comments

Manuscript number: RC-2025-03135

Corresponding author(s): Sebastian Rumpf

1. General Statements [optional]

In our manuscript, we describe a role for the nuclear mRNA export factor UAP56 (a helicase) during metamorphic dendrite and presynapse pruning in flies. We characterize a UAP56 ATPase mutant and find that it rescues the pruning defects of a uap56 mutant. We identify the actin severing enzyme Mical as a potentially crucial UAP56 mRNA target during dendrite pruning and show alterations at both the mRNA and protein level. Finally, loss of UAP56 also causes presynapse pruning defects with actin abnormalities. Indeed, the actin disassembly factor cofilin is required for pruning specifically at the presynapse.
We thank the reviewers for their constructive comments, which we tried to address experimentally as much as possible. To summarize briefly, while all reviewers saw the results as interesting (e. g., Reviewer 3's significance assessment: "Understanding how post-transcriptional events are linked to key functions in neurons is important and would be of interest to a broad audience") *and generally methodologically strong, they thought that our conclusions regarding the potential specificity of UAP56 for Mical mRNA was not fully covered by the data. To address this criticism, we added more RNAi analyses of other mRNA export factors and rephrased our conclusions towards a more careful interpretation, i. e., we now state that the pruning process is particularly sensitive to loss of UAP56. In addition, reviewer 1 had technical comments regarding some of our protein and mRNA analyses. We added more explanations and an additional control for the MS2/MCP system. Reviewers 2 and 3 wanted to see a deeper characterization of the ATPase mutant provided. We generated an additional UAP56 mutant transgene, improved our analyses of UAP56 localization, and added a biochemical control experiment. We hope that our revisions make our manuscript suitable for publication.*

2. Point-by-point description of the revisions

This section is mandatory. Please insert a point-by-point reply describing the revisions that were already carried out and included in the transferred manuscript.

Comments by reviewer 1.

Major comments

1. For Figure 4, the MS2/MCP system is not quantitative. Using this technique, it is impossible to determine how many RNAs are located in each "dot". Each of these dots looks quite large and likely corresponds to some phase-separated RNP complex where multiple RNAs are stored and/or transported. Thus, these data do not support the conclusion that Mical mRNA levels are reduced upon UAP56 knockdown. A good quantitative microscopic assay would be something like smFISH. Additinally, the localization of Mical mRNA dots to dendrites is not convincing as it looks like regions where there are dendritic swellings, the background is generally brighter.

Our response
We indeed found evidence in the literature that mRNPs labeled with the MS2/MCP or similar systems form condensates (Smith et al., JCB 2015). Unfortunately, smFISH is not established for

© 2026. Published by The Company of Biologists under the terms of the Creative Commons Attribution License (https://creativecommons.org/licenses/by/4.0/).

this developmental stage and would likely be difficult due to the presence of the pupal case. To address whether the Mical mRNPs in control and UAP56 KD neurons are comparable, we characterized the MCP dots in the respective neurons in more detail and found that their sizes did not differ significantly between control and UAP56 KD neurons. To facilitate interpretability, we also increased the individual panel sizes and include larger panels that only show the red (MCP::RFP) channel. We think these changes improved the figure. Thanks for the insight.
Changes introduced: Figure 5 (former Fig. 4): Increased panel size for MCP::RFP images, left out GFP marker for better visibility. Added new analysis of MCP::RFP dot size (new Fig. 5 I).

2. Alternatively, levels of Mical mRNA could be verified by qPCR in the laval brain following pan-neuronal UAP56 knockdown or in FACS-sorted fluorescently labeled da sensory neurons. Protein levels could be analyzed using a similar approach.

Our response
We thank the reviewer for this comment. Unfortunately, these experiments are not doable as neuron-wide UAP56 KD is lethal (see Flybase entry for UAP56). From our own experience, FACS-sorting of c4da neurons would be extremely difficult as the GFP marker fluorescence intensity of UAP56 KD neurons is weak - this would likely result in preferential sorting of subsets of neurons with weaker RNAi effects. In addition, FACS-sorting whole neurons would not discriminate between nuclear and cytoplasmic mRNA.
The established way of measuring protein content in the Drosophila PNS system is immunofluorescence with strong internal controls. In our case, we also measured Mical fluorescence intensity of neighboring c1da neurons that do not express the RNAi and show expression levels as relative intensities compared to these internal controls. This procedure rules out the influence of staining variation between samples and is used by other labs as well.

3. In Figure 5, the authors state that Mical expression could not be detected at 0 h APF. The data presented in Fig. 5C, D suggest the opposite as there clearly is some expression. Moreover, the data shown in Fig. 5D looks significantly brighter than the Orco dsRNA control and appears to localize to some type of cytoplasmic granule. So the expression of Mical does not look normal.

Our response
We thank the reviewer for this comment. In the original image in Fig. 5 C, the c4da neuron overlaps with the dendrite from a neighboring PNS neuron (likely c2da or c3da). The latter neuron shows strong Mical staining. We agree that this image is confusing and exchanged this image for another one from the same genotype.
Changes introduced: Figure 5 L (former Fig. 5 C): Exchanged panel for image without overlap from other neuron.

4. Sufficient data are not presented to conclude any specificity in mRNA export pathways. Data is presented for one export protein (UAP56) and one putative target (Mical). To adequately assess this, the authors would need to do RNA-seq in UAP56 mutants.

Our response
We thank the reviewer for this comment. To address this, we tested whether knockdown of three other mRNA export factors (NXF1, THO2, THOC5) causes dendrite pruning defects, which was not the case (new Fig. S1). While these data are consistent with specific mRNA export pathways, we agree that they are not proof. We therefore toned down our interpretation and removed the conclusion about specificity. Instead, we now use the more neutral term "increased sensibility (to loss of UAP56)".
Changes introduced: Added new Figure S1: RNAi analyses of NXF1, THO2 and THOC5 in dendrite pruning. Introduced concluding sentence at the end of first *Results* paragraph: We conclude that c4da neuron dendrite pruning is particularly sensitive to loss of UAP56. (p. 6)

5. In summary, better quantitative assays should be used in Figures 4 and 5 in order to conclude the expression levels of either mRNA or protein. In its current form, this study demonstrates the novel

© 2026. Published by The Company of Biologists under the terms of the Creative Commons Attribution License (https://creativecommons.org/licenses/by/4.0/).

finding that UAP56 regulates dendrite and presynaptic pruning, potentially via regulation of the actin cytoskeleton. However, these data do not convincingly demonstrate that UAP56 controls these processes by regulating of Mical expression and defintately not by controlling export from the nucleus.

Our response
We hope that the changes we introduced above help clarify this.

6. While there are clearly dendrites shown in Fig. 1C', the cell body is not readily identifiable. This makes it difficult to assess attachment and suggests that the neuron may be dying. This should be replaced with an image that shows the soma.

Our response
We thank the reviewer for this comment. Changes introduced: we replaced the picture in the panel with one where the cell body is more clearly visible.

7. The level of knockdown in the UAS56 RNAi and P element insertion lines should be determined. It would be useful to mention the nature of the RNAi lines (long/short hairpin). Some must be long since Dcr has been co-expressed. Another issue raised by this is the potential for off-target effects. shRNAi lines would be preferable because these effects are minimized.

Our response
We thank the reviewer for this comment. Assessment of knockdown efficiency is a control to make sure the manipulations work the way they are intended to. As mRNA isolation from Drosophila PNS neurons is extremely difficult, RNAi or mutant phenotypes in this system are controlled by performing several independent manipulations of the same gene. In our case, we used two independent RNAi lines (both long hairpins from VDRC/Bloomington and an additional insertion of the VDRC line, see Table S1) as well as a mutant P element in a MARCM experiment, i. e., a total of three independent manipulations that all cause pruning defects, and the VDRC RNAi lines do not have any predicted OFF targets (not known for the Bloomington line). If any of these manipulations would not have matched, we would have generated sgRNA lines for CRISPR to confirm.

Minor comments:

1. The authors should explain what EB1:GFP is marking when introduced in the text.

Our response
We thank the reviewer for this comment. Changes introduced: we explain the EB1::GFP assay in the panel with one where the cell body is more clearly visible.

2. The da neuron images throughout the figures could be a bit larger.

Our response
We thank the reviewer for this comment. Changes introduced: we changed the figure organization to be able to use larger panels:
- the pruning analysis of the ATPase mutations (formerly Fig. 2) is now its own figure (Figure 3).
- we increased the panel sizes of the MCP::RFP images (Figure 5 A - I, formerly Fig. 4).

Reviewer #1 (Significance (Required)):

Strengths:

The methodology used to assess dendrite and presynaptic prunings are strong and the phenotypic analysis is conclusive.

Our response
We thank the reviewer for this comment.

© 2026. Published by The Company of Biologists under the terms of the Creative Commons Attribution License (https://creativecommons.org/licenses/by/4.0/).

Weakness:

The evidence demonstrating that UAP56 regulates the expression of Mical is unconvincing. Similarly, no data is presented to show that there is any specificity in mRNA export pathways. Thus, these major conclusions are not adequately supported by the data.

Our response
We hope the introduced changes address this comment.

Reviewer #2 (Evidence, reproducibility and clarity (Required)):

In this paper, the authors describe dendrite pruning defects in c4da neurons in the DEXD box ATPase UAP56 mutant or in neuronal RNAi knockdown. Overexpression UAP56::GFP or UAP56::GFPE194Q without ATPase activity can rescue dendrite pruning defects in UAP56 mutant. They further characterized the mis-localization of UAP56::GFPE194Q and its binding to nuclear export complexes. Both microtubules and the Ubiquitin-proteasome system are intact in UAP56RNAi neurons. However, they suggest a specific effect on MICAL mRNA nuclear export shown by using the MS2-MCP system., resulting in delay of MICAL protein expression in pruned neurons. Furthermore, the authors show that UAP56 is also involved in presynaptic pruning of c4da neuros in VNC and Mica and actin are also required for actin disassembly in presynapses. They propose that UAP56 is required for dendrite and synapse pruning through actin regulation in Drosophila. Following are my comments.

Major comments
1. The result that UAP56::GFPE194Q rescues the mutant phenotype while the protein is largely mis-localized suggests a novel mechanism or as the authors suggested rescue from combination of residual activities. The latter possibility requires further support, which is important to support the role mRNA export in dendrite and pre-synapse pruning. One approach would be to examine whether other export components like REF1, and NXF1 show similar mutant phenotypes. Alternatively, depleting residual activity like using null mutant alleles or combining more copies of RNAi transgenes could help.

Our response
We thank the reviewer for this comment. We agree that the mislocalization phenotype is interesting and could inform further studies on the mechanism of UAP56. To further investigate this and to exclude that this could represent a gain-of-function due to the introduced mutation, we made and characterized a new additional transgene, UAP56::GFP E194A. This mutant shows largely the same phenotypes as E194Q, with enhanced interactions with Ref1 and partial mislocalization to the cytoplasm. In addition, we tested whether knockdown of THO2, THOC5 or NXF1 causes pruning defects (no).
Changes introduced:
- added new Figure S1: RNAi analyses of NXF1, THO2 and THOC5 in dendrite pruning.
- made and characterized a new transgene UAP56 E194A (new Fig. 2 B, E, E', 3 C, C', E, F).

2. The localization of UAP56::GFP (and E194Q) should be analyzed in more details. It is not clear whether the images in Fig. 2A and 2B are from confocal single sections or merged multiple sections. The localization to the nuclear periphery of UAP56::GFP is not clear, and the existence of the E194Q derivative in both nucleus and cytosol (or whether there is still some peripheral enrichment) is not clear if the images are stacked.

Our response
We thank the reviewer for this comment. It is correct that the profiles in the old Figure 2 were from single confocal sections from the displayed images. As it was difficult to create good average profiles with data from multiple neurons, we now introduce an alternative quantification based on categories (nuclear versus dispersed) which includes data from several neurons for each genotype, including the new E194A transgene (new Fig 3 G). Upon further inspection, the increase at the

© 2026. Published by The Company of Biologists under the terms of the Creative Commons Attribution License (https://creativecommons.org/licenses/by/4.0/).

nuclear periphery was not always visible and may have been a misinterpretation. We therefore removed this statement.

Changes introduced:

- added new quantitative analysis of UAP56 wt and E/A, E/Q mutant localization (new Fig 3 G).

3. The Ub-VV-GFP is a new reagent, and its use to detect active proteasomal degradation is by the lack of GFP signals, which could be also due to the lack of expression. The use of Ub-QQ-GFP cannot confirm the expression of Ub-VV-GFP. The proteasomal subunit RPN7 has been shown to be a prominent component in the dendrite pruning pathway (Development 149, dev200536). Immunostaining using RPN7 antibodies to measure the RPN expression level could be a direct way to address the issue whether the proteasomal pathway is affected or not.

Our response

We thank the reviewer for this comment. We agree that it is wise to not only introduce a positive control for the Ub-VV-GFP sensor (the VCP dominant-negative VCP QQ), but also an independent control. As mutants with defects in proteasomal degradation accumulate ubiquitinated proteins (see, e. g., Rumpf et al., Development 2011), we stained controls and UAP56 KD neurons with antibodies against ubiquitin and found that they had similar levels (new Fig. S3).

Changes introduced:

- added new ubiquitin immunofluorescence analysis (new Fig. S3).

4. Using the MS2/MCP system to detect the export of MICAL mRNA is a nice approach to confirm the UAP56 activity; lack of UAP56 by RNAi knockdown delays the nuclear export of MS2-MICAL mRNA. The rescue experiment by UAS transgenes could not be performed due to the UAS gene dosage, as suggested by the authors. However, this MS2-MICAL system is also a good assay for the requirement of UAP56 ATPase activity (absence in the E194Q mutant) in this process. Could authors use the MARCM (thus reduce the use of UAS-RNAi transgene) for the rescue experiment? Also, the c4da neuronal marker UAS-CD8-GFP used in Fig4 could be replaced by marker gene directly fused to ppk promoter, which can save a copy of UAS transgene. The results from the rescue experiment would test the dependence of ATPase activity in nuclear export of MICAL mRNA.

Our response

We thank the reviewer for this comment. This is a great idea but unfortunately, this experiment was not feasible due to the (rare) constraints of Drosophila genetics. The MARCM system with rescue already occupies all available chromosomes (X: FLPase, 2nd: FRT, GAL80 + mutant, 3rd: GAL4 + rescue construct), and we would have needed to introduce three additional ones (MCP::RFP and two copies of unmarked genomic MICAL-MS2, all on the third chromosome) that would have needed to be introduced by recombination. Any Drosophilist will see that this is an extreme, likely undoable project :-(

5. The UAP56 is also involved in presynaptic pruning through regulating actin assembly, and the authors suggest that Mical and cofilin are involved in the process. However, direct observation of lifeact::GFP in Mical or cofilin RNAi knockdown is important to support this conclusion.

Our response

We thank the reviewer for this comment. In response, we analyzed the lifeact::GFP patterns of control and cofilin knockdown neurons and found that loss of cofilin also leads to actin accumulation (new Fig. 7 I, J).

Changes introduced:

- new lifeact analysis (new Fig. 7 I, J).

Minor comments:

1. RNA localization is important for dendrite development in larval stages (Brechbiel JL, Gavis ER. Curr Biol. 20;18(10):745-750). Yet, the role of UAP56 is relatively specific and shown only in later-stage pruning. It would need thorough discussion.

© 2026. Published by The Company of Biologists under the terms of the Creative Commons Attribution License (https://creativecommons.org/licenses/by/4.0/).

Our response
We thank reviewer 2 for this comment. We added the following paragraph to the discussion: "UAP56 has also been shown to affect cytoplasmic mRNA localization in Drosophila oocytes (Meignin and Davis, 2008), opening up the possibility that nuclear mRNA export and cytoplasmic transport are linked. It remains to be seen whether this also applies to dendritic mRNA transport (Brechbiel and Gavis, 2008)." (p.13)

2. Could authors elaborate on the possible upstream regulators that might be involved, as described in "alternatively, several cofilin upstream regulators have been described (Rust, 2015) which might also be involved in presynapse pruning and subject to UAP56 regulation" in Discussion?

Our response
We thank reviewer 2 for this comment. In the corresponding paragraph, we cite as example now that cofilin is regulated by Slingshot phosphatases and LIM kinase (p.14).

3. In Discussion, the role of cofilin in pre- and post-synaptic processes was described. The role of Tsr/Cofilin regulating actin behaviors in dendrite branching has been described in c3da and c4da neurons (Nithianandam and Chien, 2018 and other references) should be included in Discussion.

Our response
We thank reviewer 2 for this comment. In response we tested whether cofilin is required for dendrite pruning and found that this, in contrast to Mical, is not the case (new Fig. S6). We cite the above paper in the corresponding results section (p.12).

<u>Changes introduced:</u>
- new cofilin dendrite pruning analysis (new Fig. S6).
- added cofilin reference in Results.

4. The authors speculate distinct actin structures have to be disassembled in dendrite and presynapse pruning in Discussion. What are the possible actin structures in both sites could be elaborated.

Our response
We thank reviewer 2 for this comment. In response, we specify in the Discussion: "As Mical is more effective in disassembling bundled F-actin than cofilin (Rajan et al., 2023), it is interesting to speculate that such bundles are more prevalent in dendrites than at presynapses." (p14)

Reviewer #2 (Significance (Required)):

The study initiated a genetic screen for factors involved in a dendrite pruning system and reveals the involvement of nuclear mRNA export is an important event in this process. They further identified the mRNA of the actin disassembly factor MICAL is a candidate substrate in the exporting process. This is consistent with previous finding that MICAL has to be transcribed and translated when pruning is initiated. As the presynapses of the model c4da neuron in this study is also pruned, the dependence on nuclear export and local actin remodeling were also shown. Thus, this study has added another layer of regulation (the nuclear mRNA export) in c4da neuronal pruning, which would be important for the audience interested in neuronal pruning. The study is limited for the confusing result whether ATPase activity of the exporting factor is required.

Reviewer #3 (Evidence, reproducibility and clarity (Required)):

Summary:

© 2026. Published by The Company of Biologists under the terms of the Creative Commons Attribution License (https://creativecommons.org/licenses/by/4.0/).

In the manuscript by Frommeyer, Gigengack et al. entitled "The UAP56 mRNA Export Factor is Required for Dendrite and Synapse Pruning via Actin Regulation in Drosophila" the authors surveyed a number of RNA export/processing factors to identify any required for efficient dendrite and/or synapse pruning. They describe a requirement for a general poly(A) RNA export factor, UAP56, which functions as an RNA helicase. They also study links to aspects of actin regulation.

Overall, while the results are interesting and the impact of loss of UAP56 on the pruning is intriguing, some of the data are overinterpreted as presented. The argument that UAP56 may be specific for the MICAL RNA is not sufficiently supported by the data presented. The two stories about poly(A) RNA export/processing and the actin regulation seem to not quite be connected by the data presented. The events are rather distal within the cell, making connecting the nuclear events with RNA to events at the dendrites/synapse challenging.

Our response

We thank reviewer 3 for this comment. To address this, we tested whether knockdown of three other mRNA export factors (NXF1, THO2, THOC5) causes dendrite pruning defects, which was not the case (new Fig. S1). While these data are consistent with specific mRNA export pathways, we agree that they are not proof. We therefore toned down our interpretation and removed the conclusion about specificity. Instead, we now use the more neutral term "increased sensibility (to loss of UAP56)".

We agree that it is a little hard to tie cofilin to UAP56, as we currently have no evidence that cofilin levels are affected by loss of UAP56, even though both seem to affect lifeact::GFP in a similar way (new Fig. 7 I, J). However, a dysregulation of cofilin can also occur through dysregulation of upstream cofilin regulators such as Slingshot and LIM kinase, making such a relationship possible.

Changes introduced:

- added new Figure S1: RNAi analyses of NXF1, THO2 and THOC5 in dendrite pruning.
- introduced concluding sentence at the end of first *Results* paragraph: "We conclude that c4da neuron dendrite pruning is particularly sensitive to loss of UAP56." (p. 6)
- add new lifeact::GFP analysis of cofilin KD (new Fig. I, J).
- identify potential other targets from the literature in the Discussion (Slingshot phosphatases and LIM kinase, p.14).

There are a number of specific statements that are not supported by references. See, for example, these sentences within the Introduction- "Dysregulation of pruning pathways has been linked to various neurological disorders such as autism spectrum disorders and schizophrenia. The cell biological mechanisms underlying pruning can be studied in Drosophila." The Drosophila sentence is followed by some specific examples that do include references. The authors also provide no reference to support the variant that they create in UAP56 (E194Q) and whether this is a previously characterized fly variant or based on an orthologous protein in a different system. If so, has the surprising mis-localization been reported in another system?

Our response

We thank reviewer 3 for this comment. We added the following references on pruning and disease:

1) Howes, O.D., Onwordi, E.C., 2023. The synaptic hypothesis of schizophrenia version III: a master mechanism. Mol. Psychiatry 28, 1843-1856.

2) Tang, G., et al., 2014. Loss of mTOR-dependent macroautophagy causes autistic-like synaptic pruning deficits. Neuron 83, 1131-43.

To better introduce the E194 mutations, we explain the position of the DECD motif in the Walker B domain, give the corresponding residues in the human and yeast homologues and cite papers demonstrating the importance of this residue for ATPase activity:

3) Saguez, C., et al., 2013. Mutational analysis of the yeast RNA helicase Sub2p reveals conserved domains required for growth, mRNA export, and genomic stability. RNA 19:1363-71.

4) Shen, J., et al., 2007. Biochemical Characterization of the ATPase and Helicase Activity of UAP56, an Essential Pre-mRNA Splicing and mRNA Export Factor. J. Biol. Chem. 282, P22544-22550.

© 2026. Published by The Company of Biologists under the terms of the Creative Commons Attribution License (https://creativecommons.org/licenses/by/4.0/).

We are not aware of other studies looking at the relationship between the UAP56 ATPase and its localization. Thank you for pointing this out!

Specific Comments:

Specific Comment 1: Figure 1 shows the impact of loss of UAP56 on neuron dendrite pruning. The experiment employs both two distinct dsRNAs and a MARCM clone, providing confidence that there is a defect in pruning upon loss of UAP56. As the authors mention screening against 92 genes that caused splicing defects in S2 cells, inclusion of some examples of these genes that do not show such a defect would enhance the argument for specificity with regard to the role of UAP56. This control would be in addition to the more technical control that is shown, the mCherry dsRNA.

Our response
We thank reviewer 3 for this comment. To address this, we included the full list of screened genes with their phenotypic categorization regarding pruning (103 RNAi lines targeting 64 genes) as Table S1. In addition, we also tested four RNAi lines targeting the nuclear mRNA export factors Nxf1, THO2 and THOC5 which do not cause dendrite pruning defects (Fig. S1).
Changes introduced:
- added RNAi screen results as a list in Table S1.
- added new Figure S1: RNAi analyses of NXF1, THO2 and THOC5 in dendrite pruning.

Specific Comment 2: Later the authors demonstrate a delay in the accumulation of the Mical protein, so if they assayed these pruning events at later times, would the loss of UAP56 cause a delay in these events as well? Such a correlation would enhance the causality argument the authors make for Mical levels and these pruning events.

Our response
We thank reviewer 3 for this comment. Unfortunately, this is somewhat difficult to assess, as shortly after the 18 h APF timepoint, the epidermal cells that form the attachment substrate for c4da neuron dendrites undergo apoptosis. Where assessed (e. g., Wang et al., 2017, Development) 144: 1851–1862), this process, together with the reduced GAL4 activity of our ppk-GAL4 during the pupal stage (our own observations), eventually leads to pruning, but the causality cannot be easily attributed anymore. We therefore use the 18 h APF timepoint essentially as an endpoint assay.

Specific Comment 3: Figure 2 provides data designed to test the requirement for the ATPase/helicase activity of UAP56 for these trimming events. The first observation, which is surprising, is the mislocalization of the variant (E194Q) that the authors generate. The data shown does not seem to indicate how many cells the results shown represent as a single image and trace is shown the UAP56::GFP wildtype control and the E194Q variant.

Our response
We thank reviewer 3 for this comment. It is correct that the traces shown are from single confocal sections. To better display the phenotypic penetrance, we now added a categorical analysis that shows that the UAP56 E194Q mutant is completely mislocalized in the majority of cells assessed (and the newly added E194A mutant in a subset of cells).
Changes introduced:
- added categorical quantification of UAP56 variant localization (new Fig. 2 G).

Specific Comment 4: Given the rather surprising finding that the ATPase activity is not required for the function of UAP56 characterized here, the authors do not provide sufficient references or rationale to support the ATPase mutant that they generate. The E194Q likely lies in the Walker B motif and is equivalent to human E218Q, which can prevent proper ATP hydrolysis in the yeast Sub2 protein. There is no reference to support the nature of the variant created here.

Our response

© 2026. Published by The Company of Biologists under the terms of the Creative Commons Attribution License (https://creativecommons.org/licenses/by/4.0/).

We thank reviewer 3 for this comment. To better introduce the E194 mutations, we explain the position of the DECD motif in the Walker B domain, give the corresponding residues in the human and yeast homologues (Sub2) and cite papers demonstrating the importance of this residue for ATPase activity:

1) Saguez, C., et al., 2013. Mutational analysis of the yeast RNA helicase Sub2p reveals conserved domains required for growth, mRNA export, and genomic stability. RNA 19:1363-71.
2) Shen, J., et al., 2007. Biochemical Characterization of the ATPase and Helicase Activity of UAP56, an Essential Pre-mRNA Splicing and mRNA Export Factor. J. Biol. Chem. 282, P22544-22550.

Specific Comment 5: Given the surprising results, the authors could have included additional variants to ensure the change has the biochemical effect that the authors claim. Previous studies have defined missense mutations in the ATP-binding site- K129A (Lysine to Alanine): This mutation, in both yeast Sub2 and human UAP56, targets a conserved lysine residue that is critical for ATP binding. This prevents proper ATP binding and consequently impairs helicase function. There are also missense mutations in the DEAD-box motif, (Asp-Glu-Ala-Asp) involved in ATP binding and hydrolysis. Mutations in this motif, such as D287A in yeast Sub2 (corresponding to D290A in human UAP56), can severely disrupt ATP hydrolysis, impairing helicase activity. In addition, mutations in the Walker A (GXXXXGKT) and Walker B motifs are can impair ATP binding and hydrolysis in DEAD-box helicases. Missense mutations in these motifs, like G137A (in the Walker A motif), can block ATP binding, while E218Q (in the Walker B motif)- which seems to be the basis for the variant employed here- can prevent proper ATP hydrolysis.

Our response
We thank reviewer 3 for this comment. Our cursory survey of the literature suggested that mutations in the Walker B motif are the most specific as they still preserve ATP binding and their effects have not well been characterized overall. In addition, these mutations can create strong dominant-negatives in related helicases (e. g., Rode et al., 2018 Cell Reports, our lab). To better characterize the role of the Walker B motif in UAP56, we generated and characterized an alternative mutant, UAP56 E194A. While the E194A variant does not show the same penetrance of localization phenotypes as E194Q, it also is partially mislocalized, shows stronger binding to Ref1 and also rescues the *uap56* mutant phenotypes without an obvious dominant-negative effect, thus confirming our conclusions regarding E194Q.
Changes introduced:
- added biochemical, localization and phenotypic analysis of newly generated UAP56 E194A variant (new Figs. 2 B, 2 E, E', 3 C, C'). categorical quantification of UAP56 variant localization (new Fig. 2 G).

Specific Comment 6: The co-IP results shown in Figure 2C would also seem to have multiple potential interpretations beyond what the authors suggest, an inability to disassemble a complex. The change in protein localization with the E194Q variant could impact the interacting proteins. There is no negative control to show that the UAP56-E194Q variant is not just associated with many, many proteins. Another myc-tagged protein that does not interact would be an ideal control.

Our response
We thank reviewer 3 for this comment. To address this comment, we tried to co-IP UAP56 wt or UAP56 E194Q with a THO complex subunit THOC7 (new Fig. S2). The results show that neither UAP56 variant can co-IP THOC7 under our conditions (likely because the UAP56/THO complex intermediate during mRNA export is disassembled in an ATPase-independent manner (Hohmann et al., Nature 2025)).
Changes introduced:
- added co-IP experiment between UAP56 variants and THOC7 (new Fig. S2).

Specific Comment 6: With regard to Figure 3, the authors never define EB1::GFP in the text of the Results, so a reader unfamiliar with this system has no idea what they are seeing. Reading the Materials and Methods does not mitigate this concern as there is only a brief reference to a fly line

© 2026. Published by The Company of Biologists under the terms of the Creative Commons Attribution License (https://creativecommons.org/licenses/by/4.0/).

and how the EB1::GFP is visualized by microscopy. This makes interpretation of the data presented in Figure 3A-C very challenging.

Our response
We thank reviewer 3 for pointing this out. We added a description of the EB1::GFP analysis in the corresponding Results section (p.8).

Specific Comment 7: The data shown for MICAL MS2 reporter localization in Figure 4 is nice, but is also fully expected on many former studies analyzing loss of UAP56 or UAP56 hypomorphs in different systems. While creating the reporter is admirable, to make the argument that MICAL localization is in some way preferentially impacted by loss of UAP56, the authors would need to examine several other transcripts. As presented, the authors can merely state that UAP56 seems to be required for the efficient export of an mRNA transcript, which is predicted based on dozens of previous studies dating back to the early 2000s.

Our response
Firstly, thank you for commenting on the validity of the experimental approach! The primary purpose of this experiment was to test whether the mechanism of UAP56 during dendrite pruning conforms with what is known about UAP56's cellular role - which it apparently does. We also noted that our statements regarding the specificity of UAP56 for Mical over other transcripts are difficult. While our experiments would be consistent with such a model, they do not prove it. We therefore toned down the corresponding statements (e. g., the concluding sentence at the end of first *Results* paragraphis now: "We conclude that c4da neuron dendrite pruning is particularly sensitive to loss of UAP56." (p. 6)).

Minor (and really minor) points:

In the second sentence of the Discussion, the word 'developing' seems to be mis-typed "While a general inhibition of mRNA export might be expected to cause broad defects in cellular processes, our data in develoing c4da neurons indicate that loss of UAP56 mainly affects pruning mechanisms related to actin remodeling."
Sentence in the Results (lack of page numbers makes indicating where exactly a bit tricky)- "We therefore reasoned that Mical expression could be more challenging to c4da neurons." This is a complete sentence as presented, yet, if something is 'more something'- the thing must be 'more than' something else. Presumably, the authors mean that the length of the MICAL transcript could make the processing and export of this transcript more challenging than typical fly transcripts (raising the question of the average length of a mature transcript in flies?).

Our response
Thanks for pointing these out. The typo is fixed, page numbers are added. We changed the sentence to: "Because of the large size of its mRNA, we reasoned that *MICAL* gene expression could be particularly sensitive to loss of export factors such as UAP56." (p.9) We hope this is more precise language-wise.

Reviewer #3 (Significance (Required)):

Understanding how post-transcriptional events are linked to key functions in neurons is important and would be of interest to a broad audience.

Original submission

First decision letter

MS ID#: jcs.264770

© 2026. Published by The Company of Biologists under the terms of the Creative Commons Attribution License (https://creativecommons.org/licenses/by/4.0/).

MS Title: The mRNA Export Factor UAP56 is Required for Dendrite and Synapse Pruning via Actin Regulation in Drosophila

Authors: Samuel Frommeyer; Ulrike Gigengack; Sandra Rode; Matthew Davies; Neele Wolterhoff; Sebastian Rumpf
Article Type: Review Commons Transfer

Dear Dr Rumpf,

I am happy to tell you that your manuscript has been accepted for publication in Journal of Cell Science, pending standard publication integrity checks.

Thank you for sending your manuscript to Journal of Cell Science through Review Commons.

© 2026. Published by The Company of Biologists under the terms of the Creative Commons Attribution License (https://creativecommons.org/licenses/by/4.0/).
